# Infrared Irradiation‐Assisted Solvent‐Free Pd‐Catalyzed (Hetero)aryl‐aryl Coupling via C−H Bond Activation

**DOI:** 10.1002/cssc.202101070

**Published:** 2021-07-22

**Authors:** Gianluigi Albano, Gianfranco Decandia, Maria Annunziata M. Capozzi, Nicola Zappimbulso, Angela Punzi, Gianluca M. Farinola

**Affiliations:** ^1^ Dipartimento di Chimica Università degli Studi di Bari Aldo Moro via Orabona, 4- 70125 Bari Italy; ^2^ Istituto per i Processi Chimico-Fisici CNR-IPCF Dipartimento di Chimica via Orabona 4 70125 Bari Italy

**Keywords:** C−H activation, C−C coupling, infrared irradiation, solvent-free, synthetic methods

## Abstract

The increasing attention towards environmentally friendly synthetic protocols has boosted studies directed to the development of green and sustainable methods for direct C−H bond arylation of (hetero)arenes. In this context, here the infrared (IR) irradiation‐assisted solvent‐free Pd‐catalyzed direct C−H bond arylation of (hetero)arenes was achieved. Several heteroaryl‐aryl coupling reactions were described, also involving heterocycles commonly used as building blocks for the synthesis of organic semiconductors. The reaction tolerated many functional groups on the aromatic nuclei. The IR‐irradiation as the energy source compared favorably with thermal heating and, in combination with solvent‐free conditions, provided an important contribution to the development of protocols fitting with the principles of green chemistry.

## Graphical abstract

**No solvent needed**: Pd‐catalyzed (hetero)aryl–aryl coupling via C−H bond activation is proven in solvent‐free conditions under IR irradiation as effective and convenient energy source. The reaction efficiently works on several conjugated cores and tolerates several functional groups on aryl iodides used as coupling partners. The protocol opens the way to energy‐effective solvent‐free methods in organic synthesis.

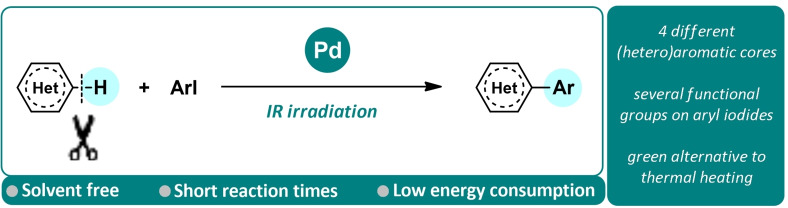

## Introduction

Green chemistry is defined as the “design of chemical products and processes to reduce or eliminate the use and generation of hazardous substances” and is based on the well‐known twelve principles, introduced in 1998 by Anastas and Warner, as operative rules to improve chemical processes sustainability.^[1]^ Over the past 20 years, the green chemistry approach enabled to redesign the organic synthesis in ways that are benign for humans and sustainable in terms of economic, social, and environmental performance. However, many challenges still lie ahead, especially in the field of carbon‐carbon bond formation.

Palladium‐catalyzed direct C−H bond arylation of (hetero)arenes^[2]^ fits well with most of the twelve principles of green chemistry, including atom economy leading to reduced wastes (principle 2), less hazardous chemical syntheses avoiding the use or generation of substances toxic to humans and/or the environment (principle 3), reduced generation of derivatives with consequent reduction of synthetic steps and minimization of additional waste (principle 8), and use of catalytic reagents (principle 9). In fact, the direct C−H bond activation eliminates the need of the preliminary preparation of air‐ and moisture‐sensitive, expensive, and toxic organometallic reagents required in traditional transition metal‐promoted cross‐coupling reactions. In a typical direct arylation process, an aryl halide reacts with the C−H bond of a (hetero)aromatic compound, in the presence of a palladium catalyst and a base to assist the C−H bond activation step, affording the corresponding coupling product.

Yet, there are aspects of direct C−H arylation processes that are scarcely compatible with the green chemistry criteria. One of the major drawbacks of most direct C−H arylation protocols is the use of toxic solvents (e. g., *N,N*‐dimethylformamide, *N*‐methyl‐2‐pyrrolidone, and *N,N*‐dimethylacetamide). Significant research efforts have been made in the last decades to set up more sustainable procedures, using environmentally benign solvents such as dialkyl carbonates, poly(ethylene glycol)s, water, γ‐valerolactone, ionic liquids, and deep eutectic solvents.[^2a^, ^2b^] According to principle 5 of green chemistry (safer solvents and auxiliaries) the development of fully solvent‐free conditions would provide even more sustainable protocols for direct arylation enabling to reduce wastes (solvent constitutes most of the mass wasted in organic synthesis), avoiding hazard and toxicity associated with some solvents and saving energy by short reaction times and simple workups. Despite these advantages, only a few examples of solvent‐free direct arylation reactions have been reported thus far.[^2a^, ^2b^, ^3^] In this context and in the frame of our studies on synthetic methods for heterocyclic‐based conjugated structures,^[4]^ we previously developed direct arylation protocols for the preparation of fully substituted 1,2,3‐triazoles^[4a]^ and extended heteroaromatic conjugated molecules,^[4b]^ performed in solvent‐free, non‐anhydrous conditions and without exclusion of air.

Until now, one of the least investigated principles of green chemistry is represented by the principle 6 (design for energy efficiency): energy requirements should be recognized for their environmental impact and should be minimized. This is a very critical point in direct C−H bond arylation reactions since high temperatures are typically required for activation of the (hetero)aryl C−H bonds. The use of non‐conventional energy sources based on microwave irradiation,^[5]^ ultrasound sonication,^[6]^ and mechanical milling^[7]^ has recently attracted attention as a valid alternative to thermal heating, leading to minimized reaction time, higher product yields, and reduced undesired by‐products.^[8]^ However, these protocols often require access to specific and expensive instruments. As an attractive alternative, infrared (IR) irradiation has been recently demonstrated to be a highly efficient form of heating emitted from inexpensive lamps (i. e., a tungsten filament sealed in a quartz envelope with a halogen gas), and it represents a promising tool for fast, cheap, and green organic synthesis by respecting the principle 6 of green chemistry. IR radiation, able to excite the molecular vibrational levels, has been used as a convenient thermal activation method for various chemical processes, such as selective extraction of natural products and condensation reactions.^[9]^ We recently reported the solvent‐free synthesis of squaraine and croconaine dyes by condensation reactions of oxocarbonic acids with indolenine‐based derivatives under IR‐light activation.^[9c]^ However, the potential of IR‐assisted reactions is still almost unexplored, especially for palladium‐catalyzed coupling chemistry. Only a few examples of palladium‐catalyzed Heck reactions^[9a]^ and Suzuki‐Miyaura cross couplings^[9b]^ promoted by IR irradiation have been reported so far.

In this work, we report the first application of IR irradiation to palladium‐catalyzed direct C−H bond arylation reactions of (hetero)arenes, which are performed under solvent‐free and non‐anhydrous conditions. Our experiments show that this protocol reduces energy consumption with respect to the conventional thermal activation of reagents. In particular, the effectiveness of the IR irradiation as energy source in the solvent‐free C−H activation reaction of several heterocyclic structures leading to the synthesis of conjugated frameworks, is demonstrated. Combining the typical features of direct C−H arylation reactions with the use of IR irradiation under solvent‐free conditions enabled the development of a highly sustainable synthetic protocol, providing a clear progress in the context of green chemistry.

## Results and Discussion

We started our study from direct arylation reaction of benzo[*b*]thiophene **1** with several aryl iodides. Arylated benzo[*b*]thiophenes are used as scaffolds in biologically active compounds and molecular semiconductors, and therefore several protocols, mostly based on the use of a Pd catalyst, have been developed for direct arylation of **1**. Although C2‐regioselectivity is commonly observed,^[10]^ C3‐arylation has been also reported under heterogeneous Pd catalysis.^[11]^ The protocols described typically require high‐boiling solvents such as *N*,*N*‐dimethylacetamide or *N*,*N*‐dimethylformamide, which are toxic, and only recently examples of room‐temperature arylation of **1** in hexafluoro‐2‐propanol^[10a]^ or water^[10d]^ have been reported. Furthermore, long reaction time is needed with a few exceptions^[12]^ including a microwave‐assisted procedure.^[12c]^ We studied the IR‐promoted arylation reaction of **1** with iodobenzene in the absence of solvent and in various experimental conditions (Table 1). Homogeneous catalysts, such as Pd_2_(dba)_3_ in the presence of P(*o*‐MeOPh)_3_, Cs_2_CO_3_, and pivalic acid (PivOH)^[4b]^ and Pd(OPiv)_2_ in the presence of P(*o*‐MeOPh)_3_ and Ag_2_CO_3_,^[4b]^ in non‐anhydrous conditions and without exclusion of air, were initially tested leading to the coupling product **2** **a** in low (25 %, entry 1) to moderate yield (61 %, entry 2), respectively. In the latter conditions higher C2‐regioselectivity was observed (89 : 11 vs. 81 : 19). Supported Pd^0^ catalysts were then investigated. **2** **a** was obtained in 25 % yield (C2/C3 ratio 90 : 10, entry 3) in the presence of Pd/C and tetra‐*n‐*butylammonium acetate (Bu_4_NOAc) as the base,^[4a]^ while reaction yield increased to 59 % (C2/C3 ratio 99 : 1, entry 4) when Pd/C was used in the presence of P(*o*‐MeOPh)_3_ and Ag_2_CO_3_. Pd/AlO(OH) nanoparticles without any additive are unable to catalyze the direct arylation of **1** (entry 5). When Pd/AlO(OH) nanoparticles at loadings as low as 0.15 mol% were used in the presence of Bu_4_NOAc or P(*o*‐MeOPh)_3_ and Ag_2_CO_3_, C2‐regioselective arylation occurred affording **2** **a** in 25 % yield (C2/C3 ratio 89 : 11, entry 6) and 65 % yield (C2/C3 ratio 98 : 2, entry 7), respectively. As it can be seen from results in entries 8 and 9, both phosphine and silver salt are needed for reaction to be proceed. The substitution of P(*o*‐MeOPh)_3_ with the less expansive PPh_3_ (entry 10) led to comparable yields (62 vs. 65 %) and a slightly higher C2‐regioselectivity (99.5 vs. 98). Similarly, it was possible to reduce PPh_3_ loading from 10 to 3 % without reduction of the yield (entry 11). Change of stoichiometry of the two coupling partners as well as of the catalyst loading was explored to improve the reaction conversion. We found that increasing the molar ratio **1**/PhI from 1 : 1 to 1 : 1.5 (entry 12) or reducing to 1.5 : 1 (entry 13) does not significantly improve the reaction yield (59 and 63 %, respectively) and C2/C3 regioselectivity, while increasing catalyst loading to 0.3 mol% negatively affects the reaction outcome lowering the reaction yield to 49 % (entry 14). Remarkably, reduction of the reaction time from 1 h (entry 11) to 15 min (entry 15) gave quite similar conversion (80 vs. 83 %), C2/C3 regioselectivity (>99 % in both cases) and yield (60 vs. 58 %), enabling significant reduction of the energy consumption.

**Table 1:** Optimization of the synthesis of **2** **a**.

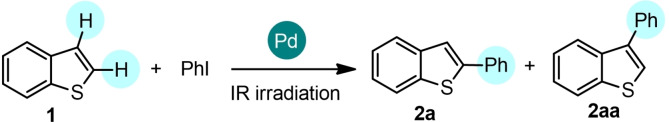								
Entry	1/PhI	Catalyst [mol %]	Phosphine [mol %]	Base (1 equiv.)	*t*	Conv.^[a]^ [%]	2a/2aa^[a]^	Yield of 2a^[b]^ [%]
1	1 : 1	Pd_2_(dba)_3_ (2)	P(*o*‐MeOPh)_3_ (4)	Cs_2_CO_3_	1 h	49	81 : 19	25^[c]^
2	1 : 1	Pd(OPiv)_2_ (5)	P(*o*‐MeOPh)_3_ (10)	Ag_2_CO_3_	1 h	86	89 : 11	61
3	1 : 1	Pd/C (5)	–	*n*Bu_4_NOAc	1 h	54	90 : 10	24
4	1 : 1	Pd/C (5)	P(*o*‐MeOPh)_3_ (10)	Ag_2_CO_3_	2 h	88	99 : 1	59
5	1 : 1	Pd/AlO(OH) (0.15)	–	*–*	1 h	0	–	–
6	1 : 1	Pd/AlO(OH) (0.15)	–	*n*Bu_4_NOAc	1 h	50	89 : 11	25
7	1 : 1	Pd/AlO(OH) (0.15)	P(*o*‐MeOPh)_3_ (10)	Ag_2_CO_3_	1 h	85	98 : 2	65
8	1 : 1	Pd/AlO(OH) (0.15)	P(*o*‐MeOPh)_3_ (10)	–	1 h	0	–	–
9	1 : 1	Pd/AlO(OH) (0.15)	–	Ag_2_CO_3_	1 h	17	71 : 29	10
10	1 : 1	Pd/AlO(OH) (0.15)	PPh_3_ (10)	Ag_2_CO_3_	1 h	82	99.5:0.5	62^[d]^
11	1 : 1	Pd/AlO(OH) (0.15)	PPh_3_ (3)	Ag_2_CO_3_	1 h	80	99.5:0.5	60
12	1 : 1.5	Pd/AlO(OH) (0.15)	PPh_3_ (3)	Ag_2_CO_3_	1 h	78	99.5:0.5	59
13	1.5 : 1	Pd/AlO(OH) (0.15)	PPh_3_ (3)	Ag_2_CO_3_	2 h	–	99.5:0.5	63
14	1 : 1	Pd/AlO(OH) (0.3)	PPh_3_ (3)	Ag_2_CO_3_	1 h	73	99 : 1	49
15	1 : 1	Pd/AlO(OH) (0.15)	PPh_3_ (3)	Ag_2_CO_3_	15 min	83	99.5:0.5	58^[e]^

[a] Conversion and C2/C3 regioselectivity by GC–MS analysis of crude reaction mixtures. [b] Unless specified, C−H arylations were carried out on 1 mmol scale. Yields on isolated product. [c] Pivalic acid (30 mol%) was used as additive. [d] After replacing Ag_2_CO_3_ with Cs_2_CO_3_, **2** **a** was isolated in 14 % yield (reaction conversion: 28 %; C2/C3 ratio: 78 : 22). [e] This reaction was repeated on 3 mmol scale (1st run: 58 % yield, 2nd run: 32 % yield).

**Scheme 1:**
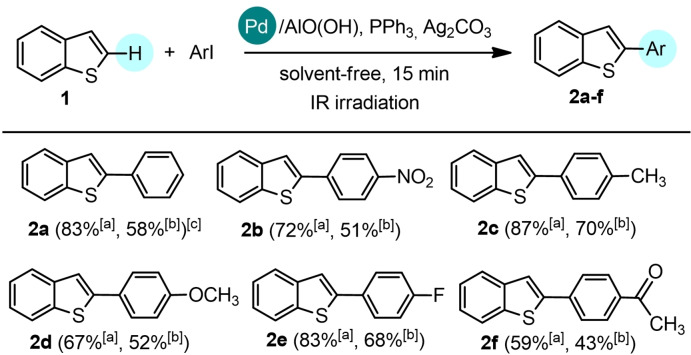
Direct arylation of benzo[*b*]thiophene **1** with iodoarenes. The C−H arylation was performed on 1 mmol scale: **1** (1 equiv.), aryl iodide (1 equiv.), Pd/AlO(OH) nanoparticles (0.15 mol%), PPh_3_ (3 mol%), Ag_2_CO_3_ (1 equiv.) in solvent‐free and non‐anhydrous conditions and in the presence of air, under IR radiation for 15 min. [a] Conversion by GC‐MS analysis. α‐Selectivity >99 % by GC‐MS analysis. [b] Isolated yield. [c] Conversion of 20 % was detected replacing iodobenzene with bromobenzene.

To investigate the scope of the reaction, **1** was reacted with various aryl iodides under the experimental conditions defined in entry 15 of Table 1. The results of the screening are shown in Scheme 1. The benzo[*b*]thiophene‐aryl coupling reactions occurred in moderate to good yields using aryl iodides functionalized with both electron‐donating functionalities, such as methyl and methoxy groups (**2** **c**: 70 % and **2** **d**: 52 %, respectively) and electron‐withdrawing groups (**2** **b**: 51 %, **2** **e**: 68 %, **2** **f**: 43 %). In the same experimental conditions, a conversion of 20 % was detected replacing iodobenzene with bromobenzene.

We also explored 5‐octylthieno[3,4‐*c*]pyrrole‐4,6‐dione (TPD) (**3**) as the starting reagent. This core is widely used as an electron‐deficient unit in the synthesis of low‐bandgap donor–acceptor molecules and polymers for organic solar cells, which can be synthesized by direct (hetero)arylation polymerization.^[13]^ Preliminary investigation was carried out using **3** with iodobenzene under different experimental conditions (Table 2).

**Table 2:** Optimization of the synthesis of **4** **a**.

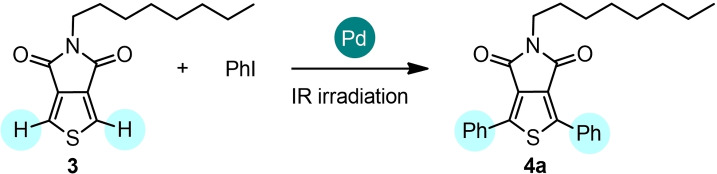							
Entry	3/PhI	Catalyst [mol %]	Phosphine [mol %]	Base (1 equiv.)	*t*	Conv.^[a]^ [%]	Yield of 4a^[a]^ [%]
1	1 : 2.4	Pd/AlO(OH) (0.3)	PPh_3_ (3)	Ag_2_CO_3_	2 h	96^[b]^	59
2	1 : 3	Pd/AlO(OH) (0.3)	PPh_3_ (3)	Ag_2_CO_3_	2 h	91^[b]^	65
3^[c]^	1 : 3	Pd_2_(dba)_3_ (2)	P(*o*‐MeOPh)_3_ (4)	Cs_2_CO_3_	2 h	96^[b]^	52
4	1 : 3	Pd(OPiv)_2_ (5)	P(*o*‐MeOPh)_3_ (10)	Ag_2_CO_3_	1 h	100^[d]^	78
5	1 : 3	Pd(OPiv)_2_ (5)	PPh_3_ (10)	Ag_2_CO_3_	1 h	100^[d]^	79
6	1 : 3	Pd(OPiv)_2_ (2)	PPh_3_ (4)	Ag_2_CO_3_	1 h	100^[d]^	77
7	1 : 3	Pd(OPiv)_2_ (1)	PPh_3_ (2)	Ag_2_CO_3_	1 h	99^[b]^	70
8	1 : 3	Pd(OPiv)_2_ (2)	PPh_3_ (4)	Ag_2_CO_3_	30 min	100^[d]^	72

[a] Reactions carried out on 0.5 mmol scale. Yields on isolated product. [b] Conversion by GC–MS analysis. [c] Pivalic acid (30 mol%) was used as additive. [d] Complete conversion by thin‐layer chromatography (TLC) and GC‐MS analysis.

When **3** was reacted with iodobenzene (**3**/PhI 1 : 2.4 molar ratio) using Pd/AlO(OH) nanoparticles as the catalyst in the presence of PPh_3_ and Ag_2_CO_3_, **4** **a** was obtained in moderate yield (59 %, entry 1) and a significant amount of mono‐coupling product was obtained. No significant increase of the yield was observed by increasing **3**/PhI molar ratio to 1 : 3 (65 %, entry 2). The yield was also moderate using Pd_2_(dba)_3_ in the presence of P(*o*‐MeOPh)_3_, Cs_2_CO_3_, and PivOH (52 %, entry 3), whereas Pd(OPiv)_2_ in the presence of P(*o*‐MeOPh)_3_ and Ag_2_CO_3_ afforded **4** **a** in 78 % (entry 4). Having selected Pd(OPiv)_2_ as the best catalyst, we examined the role of phosphine ligands, finding again that the replacement of P(*o*‐MeOPh)_3_ with the less expansive PPh_3_ led to a quite similar reaction outcome (79 % yield, entry 5). Lowering the Pd(OPiv)_2_ loading to 2 mol% (entry 6) resulted in a negligible decrease of the reaction yield (76 vs. 79 %). Further decreasing the amount of the catalyst to 1 mol% (entry 7) or shortening the reaction time to 30 min (entry 8) produced a more significant decrease of the yield to 70 % (entry 7) and 72 % (entry 8), respectively.

**Scheme 2:**
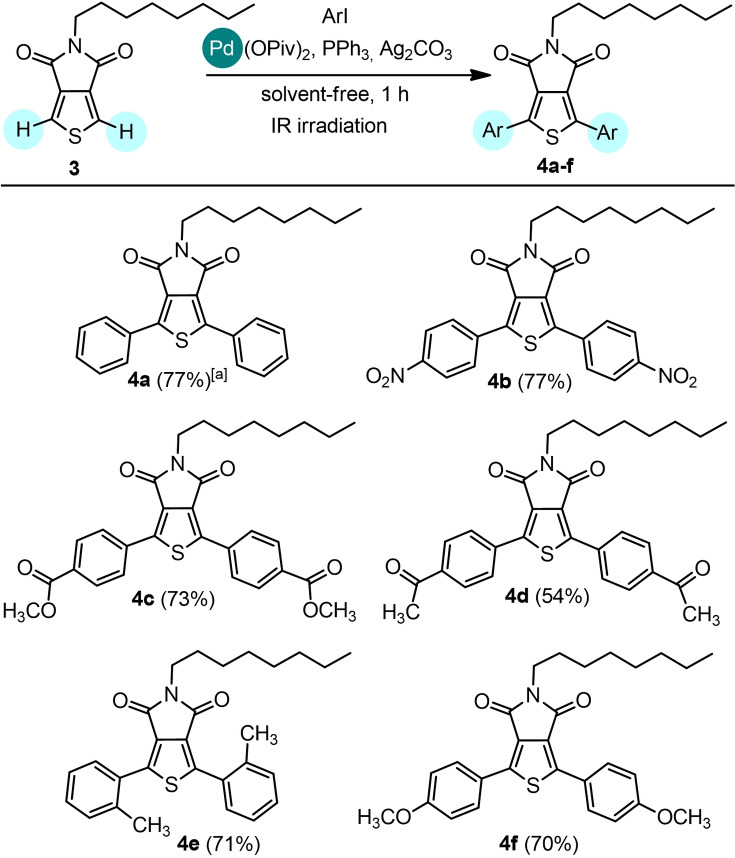
Direct arylation of 5‐octylthieno[3,4‐*c*]pyrrole‐4,6‐dione **3** with iodoarenes. The C−H arylation was performed on 0.5 mmol scale: **3** (1 equiv.), aryl iodide (3 equiv.), Pd(OPiv)_2_ (2 mol %), PPh_3_ (4 mol %), Ag_2_CO_3_ (1 equiv.) in solvent‐free and non‐anhydrous conditions and in the presence of air, under IR radiation for 1 h. Complete conversion by TLC and GC–MS analysis. Isolated yields. [a] **4** **a** was obtained in 16 % yield by use of bromobenzene instead of iodobenzene.

With the optimized conditions in hand (Table 2, entry 6), we investigated the scope of the reaction (Scheme 2). TPD **3** was coupled with a series of aryl iodides affording the bis‐arylated compounds **4** **a**–**f** in in good to excellent yields after only 1 h of IR irradiation. Both electron‐donating functionalities, such as methyl in the *ortho*‐position and methoxy groups (**4** **e**: 71 % and **4** **f**: 70 %, respectively) and electron‐withdrawing groups (**4** **b**: 77 %, **4** **c**: 73 %, **4** **d**: 54 %) can be tolerated. In the same experimental conditions, **4** **a** was obtained in 16 % yield (69 % conversion) using bromobenzene instead of iodobenzene.

1‐Hexadecyl‐4‐phenyl‐1*H*‐1,2,3‐triazole **5** was then selected as a model nitrogen aromatic heterocycle because of the relevance of triazolyl^[14]^ motif in many compounds of interest in biology and materials science. Although the Pd‐catalyzed direct arylation of 1,4‐disubstituted 1,2,3‐triazoles is the most general approach for the synthesis of fully substituted 1,2,3‐triazoles, only a few examples of sustainable direct arylation protocols based on the use of environmentally benign reaction solvents (e. g., polyethylene glycol, γ‐valerolactone) instead of the traditional high‐boiling toxic solvents (e. g., *N*,*N*‐dimethylformamide, *N*‐methyl‐2‐pyrrolidone, toluene) have been reported in the literature.^[15]^ Recently, we developed the first Pd‐catalyzed direct arylation protocol of 1,4‐disubstituted 1,2,3‐triazoles performed in solvent‐free conditions affording fully substituted 1,2,3‐triazoles in 44–81 % yields after 24 h of conventional thermal heating.^[4b]^

A preliminary screening of the experimental conditions shows that the solvent‐free direct arylation of 1,4‐disubstituted 1,2,3‐triazoles can be carried out in shorter reaction time by IR irradiation (Table 3). When **5** was reacted with iodobenzene (**5**/PhI 1 : 1.5 molar ratio) using Pd/AlO(OH) nanoparticles as the catalyst in the presence of PPh_3_ and Ag_2_CO_3_, **6** **a** was obtained in low yield (38 %, entry 1). The use of Pd(OPiv)_2_ as the catalyst in the presence of PPh_3_ and Ag_2_CO_3_ afforded **6** **a** in 69 % yield after 30 min (entry 2) and in 76 % yield after 1 h (entry 3). Increasing the **5**/PhI molar ratio to 1 : 2 and the PPh_3_ loading to 6 mol% resulted in a small decrease of the reaction yields (entries 4 and 5). On the contrary, increasing the reaction time to 2 h afforded a higher yield (83 %, entry 6).

**Table 3:** Optimization of the synthesis of **6** **a**.

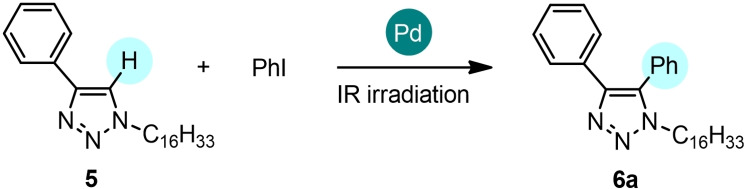							
Entry	5/PhI	Catalyst [mol %]	Phosphine [mol %]	Base (1 equiv.)	*t*	Conv.^[a]^ [%]	Yield^[b]^ [%]
1	1 : 1.5	Pd/AlO(OH) (0.15)	PPh_3_ (3)	Ag_2_CO_3_	2 h	60	38
2	1 : 1.5	Pd(OPiv)_2_ (2)	PPh_3_ (4)	Ag_2_CO_3_	30 min	86	69
3	1 : 1.5	Pd(OPiv)_2_ (2)	PPh_3_ (4)	Ag_2_CO_3_	1 h	95	76
4	1 : 2	Pd(OPiv)_2_ (2)	PPh_3_ (4)	Ag_2_CO_3_	1 h	100	71
5	1 : 1.5	Pd(OPiv)_2_ (2)	PPh_3_ (6)	Ag_2_CO_3_	1 h	97	71
6	1 : 1.5	Pd(OPiv)_2_ (2)	PPh_3_ (4)	Ag_2_CO_3_	2 h	97	83

[a] Conversion by recovery of unreacted triazole **1**. [b] Reactions carried out on 0.5 mmol scale. Yields on isolated product.

**Scheme 3:**
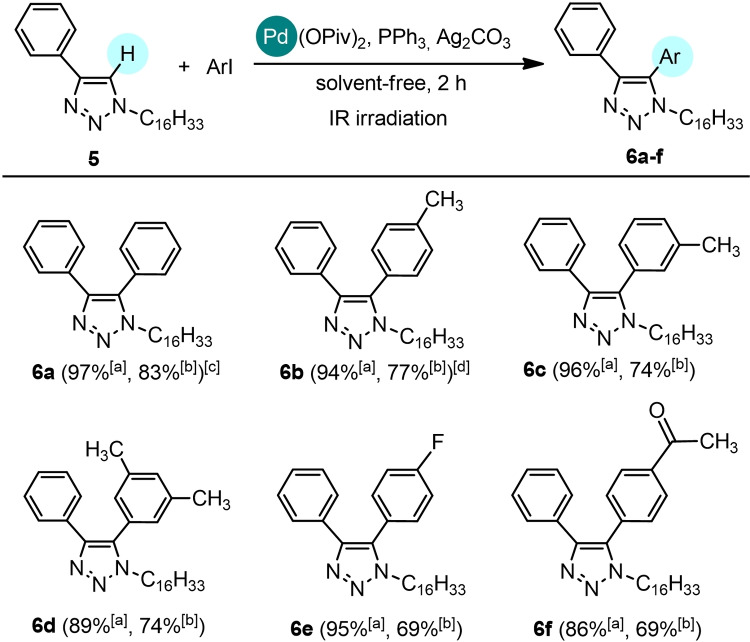
Direct arylation of 1‐hexadecyl‐4‐phenyl‐1*H*‐1,2,3‐triazole **5** with iodoarenes. The C−H arylation was performed on 0.5 mmol scale: **5** (1 equiv.), aryl iodide (1.5 equiv.), Pd(OPiv)_2_ (2 mol %), PPh_3_ (4 mol %), Ag_2_CO_3_ (1 equiv.) in solvent‐free and non‐anhydrous conditions and in the presence of air, under IR radiation for 2 h. [a] Conversion by recovery of unreacted triazole **5**. [b] Isolated yields. Reactions carried out on 0.5 mmol scale. [c] A conversion of 40 % was detected replacing iodobenzene with bromobenzene. [d] In the same experimental conditions, after 1 h: 78 % conversion, 60 % yield.

In the latter conditions, the scope of the reaction was investigated using **5** with aryl iodides bearing various substituents (Scheme 3). All the coupling reactions occurred in good yields using aryl iodides substituted with both electron‐donating functionalities, such as methyl groups (**6** **b**: 77 %, **6** **c**: 74 %, **6** **d**: 74 %) and electron‐withdrawing groups (**6** **e**: 69 %, **6** **f**: 69 %). In the same experimental conditions, the conversion dropped down to 40 % when iodobenzene was replaced with bromobenzene.

Finally, we extended the investigation to the C−H arylation to fluorinated arenes, useful building blocks for the synthesis of a variety of materials for organic optoelectronics.^[16]^ In this context, we investigated the pentafluorobenzene **7** as the starting material. Several protocols based on the use of both homogeneous^[17]^ and heterogeneous Pd catalyst^[18]^ for direct arylation of **7** have been already reported in the literature. These reactions are typically carried out in high boiling solvents such as *N*,*N*‐dimethylacetamide but an example of low‐temperature arylation of **7** in THF was also reported.^[17b]^ To the best of our knowledge, the direct arylation reaction of **7** in solvent‐free conditions has not been reported so far. A preliminary screening of experimental conditions shows that the IR irradiation allows the direct arylation of **7** in solvent free conditions in very short reaction times and in the presence of a low catalytic loading (Table 4).

**Table 4:** Optimization of the synthesis of **8** **a**.

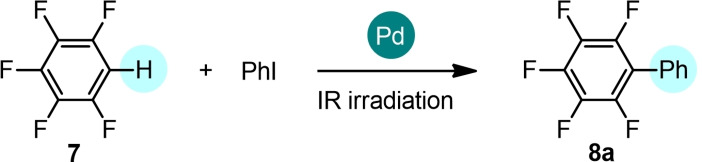						
Entry	7/PhI	Catalyst [mol %]	Phosphine [mol %]	Base (1 equiv.)	*t*	Yield^[a]^ [%]
1	1 : 1	Pd(OPiv)_2_ (2)	PPh_3_ (4)	Ag_2_CO_3_	1 h	27
2	1 : 1	Pd/AlO(OH) (0.15)	PPh_3_ (3)	Ag_2_CO_3_	1 h	85
3	1 : 1	Pd/AlO(OH) (0.15)	PPh_3_ (3)	Ag_2_CO_3_	30 min	87
4	1 : 1	Pd/AlO(OH) (0.15)	PPh_3_ (3)	Ag_2_CO_3_	15 min	87

[a] Reactions carried out on 1 mmol scale. Yields on isolated product. Complete conversion by TLC and GC‐MS analysis.

**Scheme 4:**
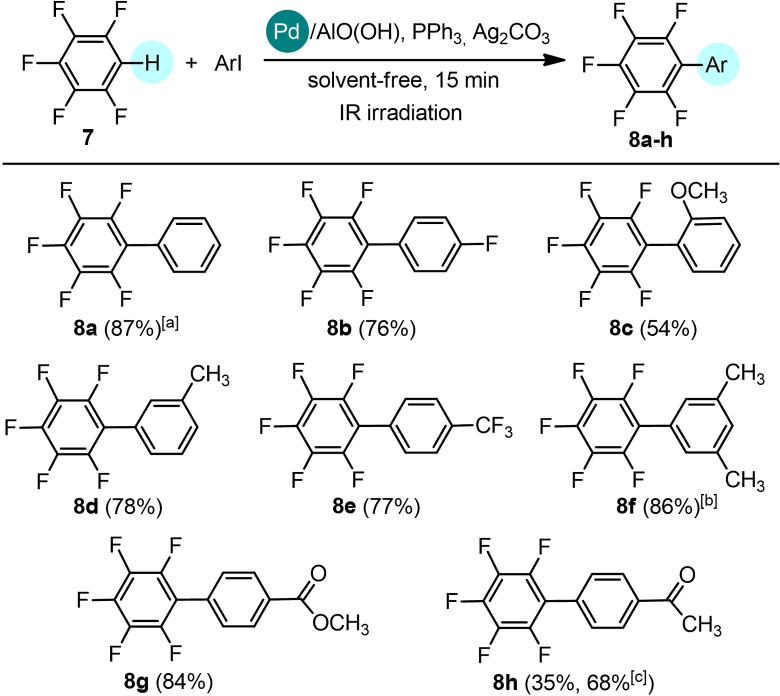
Direct arylation of pentafluorobenzene **7** with iodoarenes. The C−H arylation was performed on 1 mmol scale: **7** (1 equiv.), aryl iodide (1 equiv.), Pd/AlO(OH) (0.15 mol %), PPh_3_ (3 mol %), Ag_2_CO_3_ (1 equiv.) in solvent‐free and non‐anhydrous conditions and in the presence of air, under IR radiation for 15 min. Complete conversion by TLC and GC‐MS analysis. Isolated yields. [a] A conversion lower than 20 % was detected replacing iodobenzene with bromobenzene. [b] Reaction carried out on 0.5 mmol scale. [c] Reaction carried out in the presence of a mixture of cyclopentyl methyl ether (CPME, 0.1 mL) and dimethyl sulfoxide (DMSO, 0.1 mL).

While the expected product **8** **a** was isolated in low yield using Pd(OPiv)_2_ as the catalyst (27 %, entry 1), the use of Pd/AlO(OH) nanoparticles in only 0.15 mol% loading afforded **8** **a** in high yields (≥85 %, entries 2–4). Notably, reducing the reaction time from 1 h (entry 2) to 30 min (entry 3) and then to 15 min (entry 4) led to **8** **a** in the same yield, indicating that the reaction is very fast under these conditions. With the optimized conditions in hand (Table 4, entry 4), the scope of the reaction was investigated also in this case (Scheme 4). We found that coupling products can be obtained in good yields using aryl iodides bearing both electron‐donating functionalities, such as methyl (**8** **d**: 78 %, **8** **f**: 86 %) and methoxy (**8** **c**: 54 %) groups, and electron‐withdrawing groups (**8** **b**: 76 %, **8** **e**: 77 %, **8** **g**: 84 %, **8** **h**: 68 %). As for the other substrates, a conversion lower than 20 % was detected replacing iodobenzene with bromobenzene.

**Figure 1:**
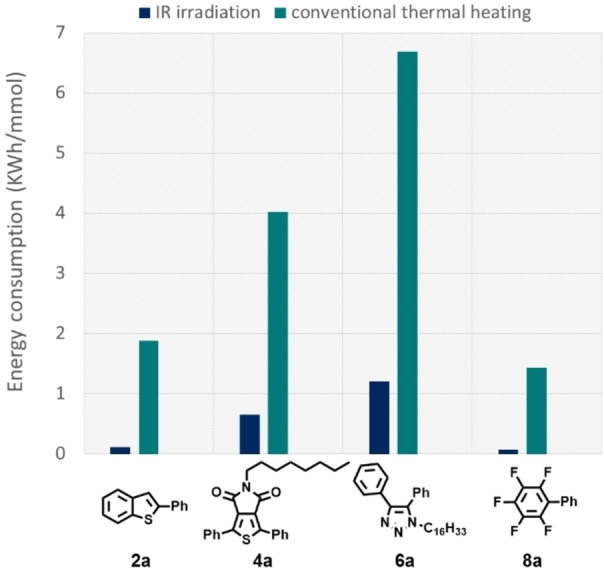
Energy consumption for the synthesis of compounds **2** **a**, **4** **a**, **6** **a**, and **8** **a** under IR irradiation and conventional thermal heating.

A comparative study of solvent‐free direct arylation protocols energy consumption under IR irradiation and under thermal heating was carried out by measuring the energy supplied to obtain one mmol of reaction product under the two different experimental conditions. For these experiments, direct arylation reactions of **1**, **3**, **5**, and **7** with iodobenzene were selected as model reactions. Each heterogeneous reaction mixture was charged in a Carius tube, then heated under IR lamp (the distance between the bottom of Carius tube and the lamp bulb was set to 7 cm) or in a pre‐warmed (160 °C) sand bath, under magnetic stirring. Keeping all the other experimental parameters the same, including reaction times, the measurements showed that the reactions performed under IR irradiation require significantly less energy compared to those performed under conventional thermal heating activation (Table 5, Figure 1). The IR‐irradiation assisted reactions afforded compounds **2** **a** and **4** **a** in yields comparable to those obtained from reactions using conventional thermal heating (**2** **a**: 58 vs. 55 %; **4** **a**: 77 vs. 82 %) with energy consumption 17 and 6 times lower, respectively. Similarly, compounds **6** **a** and **8** **a** were isolated in higher yields (**6** **a**: 83 vs. 74 %, **8** **a**: 87 vs. 72 %) with energy consumption 6 and 20 times lower, respectively. These results clearly show that IR irradiation represents an efficient and inexpensive activation method for the palladium‐catalyzed direct C−H bond arylation of (hetero)arenes in solvent‐free conditions in compliance with the principle 6 of green chemistry (design for energy efficiency).

**Table 5:** Energy consumption for the synthesis of compounds **2** **a**, **4** **a**, **6** **a**, and **8** **a** under IR irradiation and conventional thermal heating.^[a,b]^

Compound	IR irradiation^[c]^		Thermal heating^[d]^	
	Yield [%]	Energy [kWh mmol^−1^]	Yield [%]	Energy [kWh mmol^−1^]
2a	58	0.11	55	1.88
4a	77	0.65	82	4.02
6a	83	1.20	74	6.69
8a	87	0.07	72	1.43

[a] The stirred heterogeneous reaction mixture charged in a Carius tube was heated by a white IR lamp (the distance between the bottom of Carius tube and the bulb lamp was set to 7 cm) or in a pre‐warmed (160 °C) sand bath. [b] Energy consumption calculated as the energy absorption of the instrumental set up for isolated mmol of compound. [c] Energy: 0.25 kW×reaction time (h)×1/isolated mmol. [d] Energy: 0.825 kW×[pre‐heating (1 h)+reaction time (h)]×1/isolated mmol.

Finally, to further address the sustainability of our IR‐assisted conditions as a general solvent‐free protocol, we tested the combination of the solvent free arylation reaction with a product isolation protocol different from column chromatography, which requires the use of a considerable amount of solvent. As an example, the synthesis of **2** **a** was scaled‐up (reaction was carried out on 3 mmol scale) and the crude reaction mixture, after a short percolation on silica gel to eliminate catalyst and salts, was purified by crystallization from hexane. This result highlights the possibility of reducing the solvent used also in the product isolation (≈100 mL was used for both percolation and crystallization, while more than 1 L was necessary for purification on column chromatography).

## Conclusion

We have reported the first IR irradiation‐assisted, solvent‐free Pd‐catalyzed (hetero)aryl‐aryl coupling via C−H bond activation, in compliance with several principles of green chemistry. In particular, the combination of the advantages of direct C−H arylation reaction mainly related to the principles 2, 3, 8 and 9 with the use of IR irradiation (principle 6) under solvent‐free conditions (principle 5) enabled the development of a highly sustainable and environmentally friendly synthetic procedure. The protocol was tested for several (hetero)aryl‐aryl couplings, leading to diverse structural motifs. The effectiveness of the IR irradiation as a convenient energy source versus the conventional thermal heating is demonstrated. We are convinced that the application of IR irradiation to organic reactions can provide significant advantages in the context of green chemistry, which include reduced energy consumption, shortened reaction times, and even access to new mechanistic pathways, in addition to its compatibility with solvent‐free conditions as reported here. Therefore, IR irradiation may soon become a precious tool for fast, cheap, and sustainable synthesis: a powerful enabling technology which can help to usher in a greener era of organic synthesis.

## Experimental Section

### General remarks

Reagents and solvents were purchased at the highest commercial purity and used without further purification. Benzo[*b*]thiophene **1** and pentafluorobenzene **7** were purchased from Sigma Aldrich. 5‐Octylthieno[3,4‐c]pyrrole‐4,6‐dione 3 was purchased from TCI Europe and SunaTech Inc. 1‐Hexadecyl‐4‐phenyl‐1*H*‐1,2,3‐triazole **5** was prepared according to a literature procedure.^[4a]^ Preparative column chromatography was performed using Macherey‐Nagel silica gel (60, particle size 0.063–0.2 mm). Macherey‐Nagel aluminum sheets with silica gel 60 F254 were used for TLC. All new compounds were characterized by ^1^H NMR, ^13^C NMR, and LC−MS analyses. ^1^H NMR and ^13^C NMR spectra were acquired on an Agilent 500 spectrometer at 500 and at 126 MHz, respectively, using the CDCl_3_ residual proton peak at *δ*=7.26 ppm as internal standard for ^1^H spectra and the signals of CDCl_3_ at *δ*=77.16 ppm as internal standard for ^13^C spectra. GC−MS analyses were performed on a Thermo Polaris Q spectrometer equipped with a Macherey‐Nagel Optima‐1 capillary column (30 m×0.25 mm id), ionization mode EI (70 eV). Attenuated total reflectance Fourier‐transform infrared (ATR‐FTIR) spectra were acquired with a Perkin Elmer Spectrum Two Spectrophotometer equipped with A 2×2 mm Diamond crystal. Spectra were recorded in the range 4000–400 cm^−1^ with a 4 cm^−1^ resolution, using 0.25 cm^−1^ acquisition interval and acquiring 16 scans for each sample. High‐resolution mass spectra were acquired with a Shimadzu high‐performance liquid chromatography ion trap time‐of flight (LC‐ESI‐IT‐TOF) mass spectrometer via direct infusion of the samples. Melting points were determined on a Stuart Scientific Melting point apparatus SMP3. Reactions under IR‐irradiation were carried out using a Philips Infrared Industrial Heat Incandescent lamp Br125 IR 250 W 230–250 VCL 1CT (it has a broad spectrum, but most of the radiation covers the NIR with an emission peak at 1200 nm). Under IR irradiation, reaction temperatures rapidly increased up to 160 °C as detected by an IR or a digital thermometer.

### General procedure for the synthesis of compounds 2 a–f under IR irradiation

**Figure 2:**
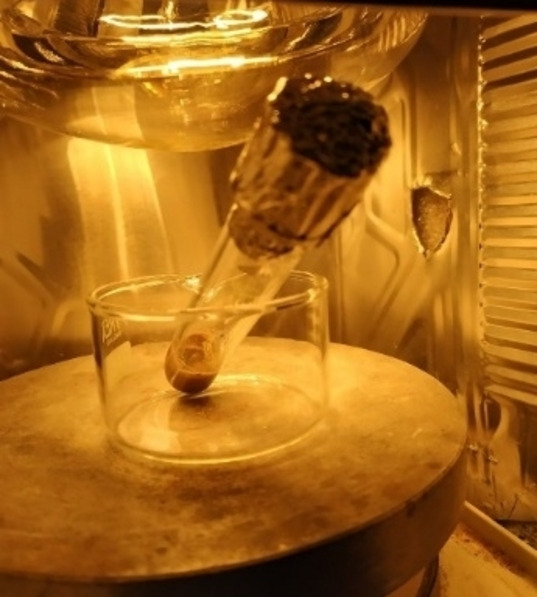
Experimental set‐up

A Carius tube (ø=1.6 cm) with a screw cap and equipped with a magnetic stirrer was charged with benzo[*b*]thiophene **1** (1.0 mmol), aryl iodide (1.0 mmol), Pd/AlO(OH) nanoparticles (0.15 mol%), PPh_3_ (3 mol%), Ag_2_CO_3_ (1.0 mmol). The resulting heterogeneous reaction mixture was heated under an IR lamp (the distance between the bottom of Carius tube and the bulb was set to 7 cm, Figure 2). After 15 min, the mixture was cooled to room temperature and the crude product was purified by column chromatography on silica gel.

**2‐Phenylbenzo[*b*]thiophene (2** **a)**:^[10a]^ Compound **2** **a** was synthesized from **1** (135 mg, 1.0 mmol) and iodobenzene (204 mg, 1.0 mmol) in accordance with the general procedure. Purification by column chromatography (*n*‐hexane/ethyl acetate=99 : 1) afforded 122 mg of compound **2** **a** as a white solid (58 % yield). Analytical data are in agreement with those previously reported in the literature.^[10a]^ *R*_f_ (*n*‐hexane/ethyl acetate=99 : 1)=0.52; ^1^H NMR (500 MHz, CDCl_3_): *δ*=7.84 (d, *J*=8.2 Hz, 1H), 7.78 (d, *J*=7.4 Hz, 1H), 7.74–7.71 (m, 2H), 7.56 (s, 1H), 7.44 (t, *J*=7.6 Hz, 2H), 7.38–7.30 ppm (m, 3H); ^13^C NMR (126 MHz, CDCl_3_): *δ*=144.4, 140.8, 139.6, 134.4, 129.1, 128.4, 126.6, 124.6, 124.4, 123.7, 122.4, 119.6 ppm; MS (70 eV): *m*/*z* (%): 210 (100) [*M*]^**+**.^, 209 (13) [*M*‐H]^+^.

**2‐(4‐Nitrophenyl)benzo[*b*]thiophene (2** **b)**:^[10a]^ Compound **2** **b** was synthesized from **1** (135 mg, 1.0 mmol) and 1‐iodo‐4‐nitrobenzene (249 mg, 1.0 mmol) in accordance with the general procedure. Purification by column chromatography (*n*‐hexane/ethyl acetate=99:1→9:1) afforded 131 mg of compound **2** **b** as a pale‐yellow solid (51 % yield). Analytical data are in agreement with those previously reported in the literature.^[10a]^ *R*_f_ (*n*‐hexane/ethyl acetate=99 : 1)=0.33; ^1^H NMR (500 MHz, CDCl_3_): *δ*=8.28 (d, *J*=9.0 Hz, 2H), 7.88–7.82 (m, 4H), 7.71 (s, 1H), 7.43–7.37 ppm (m, 2H); ^13^C NMR (126 MHz, CDCl_3_): *δ*=147.3, 141.3, 140.7, 140.4, 140.4, 126.9, 125.7, 125.2, 124.5, 124.4, 122.6, 122.6 ppm; MS (70 eV): *m*/*z* (%): 255 (100) [*M*]^**+**.^, 209 (25) [*M*‐NO_2_]^+^.

**2‐(*p*‐Tolyl)benzo[*b*]thiophene (2** **c)**:^[10a]^ Compound **2** **c** was synthesized from **1** (135 mg, 1.0 mmol) and 1‐iodo‐4‐methylbenzene (218 mg, 1.0 mmol) in accordance with the general procedure. Purification by column chromatography (*n*‐hexane) afforded 157 mg of compound **2** **c** as a white solid (70 % yield). Analytical data are in agreement with those previously reported in the literature.^[10a]^ *R*_f_ (*n*‐hexane)=0.50; ^1^H NMR (500 MHz, CDCl_3_): *δ*=7.82 (d, *J*=8.2 Hz, 1H), 7.76 (d, *J*=7.6 Hz, 1H), 7.62 (d, *J*=8.0 Hz, 2H), 7.51 (s, 1H), 7.35 (td, *J*=7.6, 1.2 Hz, 1H), 7.32–7.28 (m, 1H), 7.24 (d, *J*=8.0 Hz, 2H), 2.40 ppm (s, 3H); ^13^C NMR (126 MHz, CDCl_3_): *δ*=144.5, 140.9, 139.5, 138.4, 131.6, 129.8, 126.5, 124.6, 124.3, 123.5, 122.4, 119.0, 21.4 ppm; MS (70 eV): *m*/*z* (%): 224 (100) [*M*]^**+**.^, 223 (45) [*M*‐H]^+^.

**2‐(4‐Methoxyphenyl)benzo[*b*]thiophene (2** **d)**:^[10a]^ Compound **2** **d** was synthesized from **1** (135 mg, 1.0 mmol) and 1‐iodo‐4‐methoxybenzene (234 mg, 1.0 mmol) in accordance with the general procedure. Purification by column chromatography (*n*‐hexane/CH_2_Cl_2_=4 : 1) afforded 125 mg of compound **2** **d** as a white solid (52 % yield). Analytical data are in agreement with those previously reported in the literature.^[10a]^ *R*_f_ (*n*‐hexane/CH_2_Cl_2_=4 : 1)=0.49; ^1^H NMR (500 MHz, CDCl_3_): *δ*=7.81 (d, *J*=8.2 Hz, 1H), 7.74 (d, *J*=7.7 Hz, 1H), 7.65 (d like, *J*=8.8 Hz, 2H), 7.43 (s, 1H), 7.34 (td, *J*=7.7, 1.2 Hz, 1H), 7.31–7.26 (m, 1H), 6.96 (d like, *J*=8.8 Hz, 2H), 3.86 ppm (s, 3H); ^13^C NMR (126 MHz, CDCl_3_): *δ*=159.9, 144.3, 141.0, 139.3,127.9, 127.2, 124.6, 124.1, 123.4, 122.3, 118.4, 114.5, 55.5 ppm; MS (70 eV): *m*/*z* (%): 240 (100) [*M*]^**+**.^, 225 (93) [*M−C*H_3_]^+^.

**2‐(4‐Fluorophenyl)benzo[*b*]thiophene (2** **e)**:^[10a]^ Compound **2** **e** was synthesized from **1** (135 mg, 1.0 mmol) and 1‐fluoro‐4‐iodobenzene (222 mg, 1.0 mmol) in accordance with the general procedure. Purification by column chromatography (*n*‐hexane) afforded 155 mg of compound **2** **e** as a white solid (68 % yield). Analytical data are in agreement with those previously reported in the literature.^[10a]^ *R*_f_ (*n*‐hexane)=0.42; ^1^H NMR (500 MHz, CDCl_3_): *δ*=7.83 (d, *J*=7.8 Hz, 1H), 7.77 (d, *J*=7.6 Hz, 1H), 7.71–7.65 (m 2H), 7.47 (s, 1H), 7.36 (td, *J*=7.6, 1.2 Hz, 1H), 7.32 (td, *J*=7.8, 1.3 Hz, 1H), 7.13 ppm (t like, *J*=8.6 Hz, 2H); ^13^C NMR (126 MHz, CDCl_3_): *δ*=163.9 (d, *J*=248.4 Hz), 143.2, 140.8, 139.6, 130.7 (d, *J*=3.5 Hz), 128.3 (d, *J*=8.1 Hz), 124.8, 124.5, 123.7, 122.4, 119.6 (d, *J*=1.3 Hz), 116.1 ppm (d, *J*=21.9 Hz); MS (70 eV): *m*/*z* (%): 228 (100) [*M*]^**+**.^, 227 (9) [*M*‐H]^+^.

**1‐(4‐(Benzo[*b*]thiophen‐2‐yl)phenyl)ethanone (2** **f)**:^[10a]^ Compound **2** **f** was synthesized from **1** (135 mg, 1.0 mmol) and 1‐(4‐iodophenyl)ethanone (246 mg, 1.0 mmol) in accordance with the general procedure. Purification by column chromatography (*n*‐hexane/ethyl acetate=9 : 1) afforded 108 mg of compound **2** **f** as a white solid (43 % yield). Analytical data are in agreement with those previously reported in the literature.^[10a]^ *R*_f_ (*n*‐hexane/ethyl acetate=9 : 1)=0.29; ^1^H NMR (500 MHz, CDCl_3_): *δ*=8.02 (d like, *J*=8.5 Hz, 2H), 7.87–7.84 (m, 1H), 7.83–7.79 (m, 3H), 7.68 (s, 1H), 7.40–7.33 (m, 2H), 2.64 ppm (s, 3H); ^13^C NMR (126 MHz, CDCl_3_) *δ* :197.4, 142.8, 140.6, 140.0, 138.8, 136.5, 129.2, 126.5, 125.2, 124.9, 124.1, 122.5, 121.3, 26.8 ppm; MS (70 eV): *m*/*z* (%): 252 (65) [*M*]^**+**.^, 237 (100) [*M−C*H_3_]^+^. 209 (33) [*M−C*(O)CH_3_]^+^.

### General procedure for the synthesis of compounds 4 a–f under IR irradiation

A Carius tube (ø=1.6 cm) with a screw cap and equipped with a magnetic stirrer was charged with 5‐octylthieno[3,4‐*c*]pyrrole‐4,6‐dione **3** (0.5 mmol), aryl iodide (1.5 mmol), Pd(OPiv)_2_ (2 mol%), PPh_3_ (4 mol%), Ag_2_CO_3_ (0.5 mmol). The resulting heterogeneous reaction mixture was heated under an IR lamp (the distance between the bottom of Carius tube and the bulb was set to 7 cm, Figure 2). After 1 h, the mixture was cooled to room temperature and the crude product was purified by column chromatography on silica gel.

**5‐Octyl‐1,3‐diphenyl‐4*H*‐thieno[3,4‐*c*]pyrrole‐4,6(5*H*)‐dione (4** **a)**:[^4b^, ^4c^] Compound **4** **a** was synthesized from **3** (133 mg, 0.5 mmol) and iodobenzene (306 mg, 1.5 mmol) in accordance with the general procedure. Purification by column chromatography (*n*‐hexane/ethyl acetate=9 : 1) afforded 160 mg of compound **4** **a** as a pale‐yellow solid (77 % yield). Analytical data are in agreement with those previously reported in the literature.^[4c]^ *R*_f_ (*n*‐hexane/ethyl acetate=9 : 1)=0.68; ^1^H NMR (500 MHz, CDCl_3_): *δ*=8.13 (d like, *J*=7.0 Hz, 4H), 7.51–7.40 (m, 6H), 3.67 (t, *J*=7.5 Hz, 2H), 1.71–1.64 (m 2H), 1.39–1.22 (m, 10H), 0.86 ppm (t, *J*=7.0 Hz, 3H); ^13^C NMR (126 MHz, CDCl_3_): *δ*=162.9, 144.9, 130.6, 130.5, 130.1, 129.0, 128.0, 38.7, 31.9, 29.3, 29.3, 28.6, 27.1, 22.7, 14.2 ppm; MS (70 eV): *m*/*z* (%): 417 (91) [*M*]^**+**.^, 318 (93) [*M−C*_7_H_15_]^+^, 304 (13) [*M−C*_8_H_17_]^+^.

**1,3‐Bis(4‐nitrophenyl)‐5‐octyl‐4*H*‐thieno[3,4‐*c*]pyrrole‐4,6(5*H*)‐dione (4** **b)**:[^4b^, ^4c^] Compound **4** **b** was synthesized from **3** (133 mg, 0.50 mmol) and 1‐iodo‐4‐nitrobenzene (374 mg, 1.50 mmol), in accordance with the general procedure. Purification by column chromatography (*n*‐hexane/ethyl acetate=8:2→7:3) afforded compound **4** **g** (184 mg, 77 % yield) as a yellow solid. Analytical data are in agreement with those previously reported in the literature.^[4c]^ *R*_f_ (*n*‐hexane/ethyl acetate=8 : 2)=0.67; ^1^H NMR (500 MHz, CDCl_3_): *δ*=8.35 (s, 8H), 3.71 (t, *J*=7.2 Hz, 2H), 1.74–1.66 (m, 2H), 1.40–1.22 (m, 10H), 0.87 ppm (t, *J*=6.9 Hz, 3H); ^13^C NMR (126 MHz, CDCl_3_): *δ*=162.4, 148.5, 143.0, 135.9, 133.4, 129.2, 124.5, 39.2, 31.9, 29.3, 29.3, 28.5, 27.1, 22.8, 14.2 ppm.

**Dimethyl 4,4′‐(5‐Octyl‐4,6‐dioxo‐5,6‐dihydro‐4*H*‐thieno[3,4‐*c*]‐pyrrole‐1,3‐diyl)dibenzoate (4** **c)**:[^4b^, ^4c^] Compound **4** **c** was synthesized from **3** (133 mg, 0.50 mmol) and methyl 4‐iodobenzoate (393 mg,1.50 mmol) in accordance with the general procedure. Purification by column chromatography (*n*‐hexane/ethyl acetate=8.5 : 1.5) afforded compound **4** **c** (194 mg, 73 % yield) as a yellow solid. Analytical data are in agreement with those previously reported in the literature.^[4c]^ *R*_f_ (*n*‐hexane/ethyl acetate=8.5 : 1.5)=0.36; ^1^H NMR (500 MHz, CDCl_3_): *δ*=8.24‐8.21 (m, 4H), 8.15–8.12 (m, 4H), 3.96 (s, 6H), 3.69 (t, *J*=7.5 Hz, 2H), 1.72–1.65 (m, 2H), 1.39–1.22 (m, 10H), 0.87 ppm (t, *J*=6.9 Hz, 3H); ^13^C NMR (126 MHz, CDCl_3_): *δ*=166.3, 162.6, 144.0, 134.3, 132.1, 131.4, 130.3, 128.1, 52.5, 38.9, 31.9, 29.3, 29.3, 28.5, 27.1, 22.7, 14.2 ppm.

**1,3‐Bis(4‐acetylphenyl)‐5‐octyl‐4*H*‐thieno[3,4‐*c*]pyrrole‐4,6(5*H*)‐dione (4** **d)**:[^4b^, ^4c^] Compound **4** **d** was synthesized from **3** (133 mg, 0.5 mmol) and 1‐(4‐iodophenyl)ethanone (369 mg, 1.5 mmol) in accordance with the general procedure. Purification by column chromatography (*n*‐hexane/ethyl acetate=8 : 2) afforded compound **4** **d** (136 mg, 54 % yield) as a yellow solid. Analytical data are in agreement with those previously reported in the literature.^[4c]^ *R*_f_ (*n*‐hexane/ethyl acetate=8 : 2)=0.43; ^1^H NMR (500 MHz, CDCl_3_): *δ*=8.27–8.23 (m, 4H), 8.08–8.05 (m, 4H), 3.69 (t, *J*=7.5 Hz, 2H), 2.65 (s, 6H), 1.72–1.64 (m, 2H), 1.39–1.22 (m, 10H), 0.87 ppm (t, *J*=7.0 Hz, 3H); ^13^C NMR (126 MHz, CDCl_3_): *δ*=197.1, 162.6, 144.0, 137.9, 134.4, 132.2, 129.0, 128.3, 38.9, 31.9, 29.3, 29.3, 28.5, 27.1, 26.8, 22.7, 14.2 ppm.

**5‐Octyl‐1,3‐di‐*o–*tolyl‐4*H*‐thieno[3,4‐*c*]pyrrole‐4,6(5*H*)‐dione (4** **e)**:[^4b^, ^4c^] Compound **4** **e** was synthesized from **3** (133 mg, 0.50 mmol) and 2‐iodotoluene (327 mg, 1.50 mmol) in accordance with the general procedure. Purification by column chromatography (*n*‐hexane/ethyl acetate=9 : 1) afforded 159 mg of compound **4** **e** (71 % yield) as a pale‐yellow viscous liquid. Analytical data are in agreement with those previously reported in the literature.^[4c]^ *R*_f_ (*n*‐hexane/ethyl acetate=9 : 1)=0.61; ^1^H NMR (500 MHz, CDCl_3_): *δ*=7.51 (d, *J*=7.4 Hz, 2H), 7.39–7.32 (m, 4H), 7.30–7.26 (m, 2H), 3.57 (t, *J*=7.2 Hz, 2H), 2.48 (s, 6H), 1.66–1.58 (m, 2H), 1.32–1.19 (m, 10H), 0.85 ppm (t, *J*=7.0 Hz, 3H); ^13^C NMR (126 MHz, CDCl_3_) *δ* : 162.8, 144.5, 137.3, 131.6, 131.0, 130.9, 129.9, 129.5, 126.0, 38.4, 31.9, 29.2, 28.5, 27.0, 22.7, 20.6, 14.2 ppm (one coincident signal not observed); MS (70 eV): *m*/*z* (%): 445 (100) [*M*]^**+**.^, 430 (27) [*M−C*H_3_]^+^, 346 (98) [*M−C*_7_H_15_]^+^, 332 (75) [M−C_8_H_17_]^+^.

**1,3‐Bis(4‐methoxyphenyl)‐5‐octyl‐4*H*‐thieno[3,4‐*c*]pyrrole‐4,6(5*H*)‐dione (4** **f)**:[^4b^, ^4c^] Compound **4** **f** was synthesized from **3** (133 mg,0.5 mmol) and 1‐iodo‐4‐methoxybenzene (351 mg, 1.5 mmol) in accordance with the general procedure. Purification by column chromatography (CH_2_Cl_2_/*n*‐hexane=6 : 4) afforded compound **4** **f** (167 mg, 70 % yield) as a pale yellow solid. Analytical data are in agreement with those previously reported in the literature.^[4c]^ *R*_f_ (CH_2_Cl_2_/*n*‐hexane=6 : 4)=0.52; ^1^H NMR (500 MHz, CDCl_3_): *δ*=8.12–8.08 (m, 4H), 7.00–6.96 (m, 4H), 3.87 (s, 6H), 3.65 (t, *J*=7.5 Hz, 2H), 1.70–1.63 (m, 2H), 1.37–1.21 (m, 10H), 0.86 ppm (t, *J*=7.0 Hz, 3H); ^13^C NMR (126 MHz, CDCl_3_): *δ*=163.3, 161.1, 144.3, 129.8, 129.0, 123.7, 114.3, 55.5, 38.7, 31.9, 29.4, 29.3, 28.6, 27.1, 22.8, 14.2 ppm.

### General procedure for the synthesis of compounds 6 a–f under IR irradiation

A Carius tube (ø=1.6 cm) with a screw cap and equipped with a magnetic stirrer was charged with 1‐hexadecyl‐4‐phenyl‐1*H*‐1,2,3‐triazole **5** (0.5 mmol), aryl iodide (0.75 mmol), Pd(OPiv)_2_ (2 mol%), PPh_3_ (4 mol%), Ag_2_CO_3_ (0.5 mmol). The resulting heterogeneous reaction mixture was heated under an IR lamp (the distance between the bottom of Carius tube and the bulb was set to 7 cm, Figure 2). After 2 h, the mixture was cooled to room temperature and the crude product was purified by column chromatography on silica gel.

**1‐Hexadecyl‐4,5‐diphenyl‐1*H*‐1,2,3‐triazole (6** **a)**:^[4a]^ Compound **6** **a** was synthesized from **5** (185 mg, 0.5 mmol) and iodobenzene (153 mg, 0.75 mmol) in accordance with the general procedure. Purification by column chromatography (*n*‐hexane/ethyl acetate=8.5 : 1.5) afforded compound **6** **a** (184 mg, 83 % yield) as a white solid. Analytical data are in agreement with those previously reported in the literature.^[4a]^ *R*_f_ (*n*‐hexane/ethyl acetate=8.5 : 1.5)=0.59; ^1^H NMR (500 MHz, CDCl_3_): *δ*=7.54 (dd like, *J*=8.2, 1.7 Hz, 2H), 7.53–7.50 (m, 3H), 7.35–7.31 (m, 2H), 7.28–7.21 (m, 3H), 4.19 (t, *J*=7.2 Hz, 2H), 1.81–1.74 (m, 2H), 1.31–1.17 (m, 26H), 0.88 ppm (t, *J*=7.0 Hz, 3H); ^13^C NMR (126 MHz, CDCl_3_): *δ*=144.2, 133.8, 131.1, 130.1, 129.8, 129.5,128.5, 128.4,127.7, 126.9, 48.4, 32.1, 30.2, 29.8, 29.8, 29.8, 29.8, 29.8, 29.7, 29.6, 29.5, 29.4, 29.0, 26.5, 22.8, 14.3 ppm; MS (70 eV): *m*/*z* (%): 445 (4) [*M*]^**+**.^, 417 (2) [*M*‐N_2_]^**+**.^, 193 (100) [*M−C*_16_H_32_N_2_]^**+**.^, 192 (26) [*M−C*_16_H_33_N_2_]^+^.

**1‐Hexadecyl‐4‐phenyl‐5‐(*p–*tolyl)‐1*H*‐1,2,3‐triazole (6** **b)**:^[4a]^ Compound **6** **b** was synthesized from **5** (185 mg, 0.5 mmol) and 1‐iodo‐4‐methylbenzene (164 mg, 0.75 mmol) in accordance with the general procedure. Purification by column chromatography (*n*‐hexane/ethyl acetate=8.5 : 1.5) afforded compound **6** **b** (177 mg, 77 % yield) as a white solid. Analytical data are in agreement with those previously reported in the literature.^[4a]^ *R*_f_ (*n*‐hexane/ethyl acetate=8.5 : 1.5)=0.42; ^1^H NMR (500 MHz, CDCl_3_): *δ*=7.57–7.54 (m, 2H), 7.31 (d, *J*=8.0 Hz, 2H), 7.28–7.22 (m, 3H), 7.20 (d, *J*=8.0 Hz, 2H), 4.18 (t, *J*=7.5 Hz, 2H), 2.45 (s, 3H), 1.81–1.74 (m, 2H), 1.31–1.17 (m, 26H), 0.88 ppm (t, *J*=7.0 Hz, 3H); ^13^C NMR (126 MHz, CDCl_3_): *δ*=144.0, 139.9, 133.9, 131.1, 130.2, 129.9, 128.5, 127.7, 126.9, 125.1, 48.4, 32.1, 30.2, 29.8, 29.8, 29.8, 29.8, 29.7, 29.6, 29.5, 29.5, 29.0, 26.5, 22.8, 21.6, 14.3 ppm (one coincident signal not observed); MS (70 eV): *m*/*z* (%): 459 (4) [*M*]^**+**.^, 431 (4) [*M*‐N_2_]^**+**.^, 207 (100) [*M−C*_16_H_32_N_2_]^**+**.^, 206 (16) [*M*−C_16_H_33_N_2_]^+^.

**1‐Hexadecyl‐4‐phenyl‐5‐(*m–*tolyl)‐1*H*‐1,2,3‐triazole (6** **c)**:^[4a]^ Compound **6** **c** was synthesized from **5** (185 mg, 0.5 mmol) and 1‐iodo‐3‐methylbenzene (164 mg, 0.75 mmol) in accordance with the general procedure. Purification by column chromatography (*n*‐hexane/ethyl acetate=8.5 : 1.5) afforded compound **6** **c** (170 mg, 74 % yield) as a white solid. Analytical data are in agreement with those previously reported in the literature.^[4a]^ *R*_f_ (*n*‐hexane/ethyl acetate=8.5 : 1.5)=0.42; ^1^H NMR (500 MHz, CDCl_3_): *δ*=7.57–7.54 (m, 2H), 7.39 (t, *J*=7.9 Hz, 1H), 7.32 (br d, *J*=7.9 Hz, 1H), 7.29–7.21 (m, 3H), 7.13–7.10 (m, 2H), 4.18 (t, *J*=7.3 Hz, 2H), 2.40 (s, 3H), 1.81–1.74 (m, 2H), 1.31–1.17 (m, 26H), 0.88 ppm (t, *J*=7.0 Hz, 3H); ^13^C NMR (126 MHz, CDCl_3_): *δ*=144.0, 139.3, 134.0, 131.1, 130.6, 130.5, 129.3, 128.5, 128.2, 127.7, 127.2, 126.8, 48.4, 32.1, 30.2, 29.8, 29.8, 29.8, 29.8, 29.8, 29.7, 29.6, 29.5, 29.4, 29.0, 26.5, 22.8, 21.5, 14.2 ppm; MS (70 eV): *m*/*z* (%): 459 (5) [*M*]^**+**.^, 431 (4) [*M*‐N_2_]^**+**.^, 207 (100) [*M−C*_16_H_32_N_2_]^**+**.^, 206 (20) [*M*−C_16_H_33_N_2_]^+^.

**5‐(3,5‐Dimethylphenyl)‐1‐hexadecyl‐4‐phenyl‐1*H*‐1,2,3‐triazole (6** **d)**:^[4a]^ Compound **6** **d** was synthesized from **5** (185 mg, 0.5 mmol) and 1‐iodo‐3,5‐dimethylbenzene (174 mg, 0.75 mmol) in accordance with the general procedure. Purification by column chromatography (*n*‐hexane/ethyl acetate=8.5 : 1.5) afforded compound **6** **d** (175 mg, 74 % yield) as a white solid. Analytical data are in agreement with those previously reported in the literature.^[4a]^ *R*_f_ (*n*‐hexane/ethyl acetate=8.5 : 1.5)=0.47; ^1^H NMR (500 MHz, CDCl_3_): *δ*=7.59–7.56 (m, 2H), 7.29–7.21 (m, 3H), 7.13 (br s, 1H), 6.92 (br s, 2H), 4.16 (t, *J*=7.5 Hz, 2H), 2.36 (s, 6H), 1.82–1.75 (m, 2H), 1.31–1.17 (m, 26H), 0.88 ppm (t, *J*=7.0 Hz, 3H); ^13^C NMR (126 MHz, CDCl_3_): *δ*=143.9, 139.1, 134.1, 131.4, 131.3, 128.5, 128.1, 127.7, 127.6, 126.7, 48.3, 32.1, 30.2, 29.8, 29.8, 29.8, 29.7, 29.6, 29.5, 29.4, 29.0, 26.5, 22.8, 21.4, 14.3 ppm (two coincident signals not observed); MS (70 eV): *m*/*z* (%): 473 (5) [*M*]^**+**.^, 445 (6) [*M*‐N_2_]^**+**.^, 221 (100) [*M*−C_16_H_32_N_2_]^**+**.^, 220 (15) [*M*−C_16_H_33_N_2_]^+^.

**5‐(4‐Fluorophenyl)‐1‐hexadecyl‐4‐phenyl‐1*H*‐1,2,3‐triazole (6** **e)**: Compound **6** **e** was synthesized from **5** (185 mg, 0.5 mmol) and 1‐fluoro‐4‐iodobenzene (167 mg, 0.75 mmol) in accordance with the general procedure. Purification by column chromatography (*n*‐hexane/ethyl acetate=8 : 2) afforded compound **6** **e** (161 mg, 69 % yield) as a white solid, m.p. 65.0–66.0 °C (after crystallization from *n*‐hexane). *R*_f_ (*n*‐hexane/ethyl acetate=8 : 2)=0.69; ^1^H NMR (500 MHz, CDCl_3_): *δ*=7.52 (dd like, *J*=8.1, 1.6 Hz, 2H), 7.34–7.20 (m, 7H), 4.18 (t, *J*=7.2 Hz, 2H), 1.81–1.74 (m, 2H), 1.31–1.18 (m, 26H), 0.87 ppm (t, *J*=7.0 Hz, 3H); ^13^C NMR (126 MHz, CDCl_3_): *δ*=163.5 (d, *J*=250.7 Hz), 144.4, 132.7, 132.1 (d, *J*=8.4 Hz), 130.9, 128.6, 127.9, 126.9, 124.3 (d, *J*=3.6 Hz), 116.8 (d, *J*=21.8 Hz), 48.4, 32.1, 30.2, 29.8, 29.8, 29.8, 29.8, 29.8, 29.7, 29.6, 29.5, 29.4, 29.0, 26.5, 22.8, 14.3 ppm; IR (neat): *ṽ*=2916 (vs), 2847 (vs), 1515 (m), 1486 (M), 1471 (m), 1227 cm^−1^ (m); MS (70 eV): *m*/*z* (%): 463 (4) [*M*]^**+**.^, 435 (2) [*M*‐N_2_]^**+**.^, 211 (100) [*M*−C_16_H_32_N_2_]^**+**.^, 210 (16) [*M*−C_16_H_33_N_2_]^+^; HRMS (LC–IT‐TOF, elution with 0.1 % (*v*/*v*) formic acid in methanol) *m*/*z*: [*M*+H]^+^ Calcd for C_30_H_43_FN_3_ 464.3436; Found 464.3445, mass error=1.94 ppm, C (30 : 11).

**1‐(4‐(1‐Hexadecyl‐4‐phenyl‐1*H*‐1,2,3‐triazol‐5‐yl)phenyl)ethenone (6** **f)**: Compound **6** **f** was synthesized from **5** (185 mg, 0.5 mmol) and 1‐(4‐iodophenyl)ethanone (185 mg, 0.75 mmol) in accordance with the general procedure. Purification by column chromatography (*n*‐hexane/ethyl acetate=8.5 : 1.5→8:2) afforded compound **6** **f** (169 mg, 69 % yield) as a white solid, m.p. 57.9–58.3 °C (after crystallization from *n*‐hexane). *R*_f_ (*n*‐hexane/ethyl acetate=8.5 : 1.5)=0.22; ^1^H NMR (500 MHz, CDCl_3_): *δ*=8.09 (d like, *J*=8.4 Hz, 2H), 7.51–7.48 (m, 2H), 7.44 (d like, *J*=8.4 Hz, 2H), 7.30–7.24 (m, 3H), 4.22 (t, *J*=7.3 Hz, 2H), 2.68 (s, 3H), 1.81–1.74 (m, 2H), 1.31–1.17 (m, 26H), 0.87 ppm (t, *J*=7.0 Hz, 3H); ^13^C NMR (126 MHz, CDCl_3_): *δ*=197.4, 144.7, 137.9, 133.1, 132.7, 130.6, 130.4, 129.3, 128.7, 128.1, 127.1, 48.7, 32.0, 30.2, 29.8, 29.8, 29.8, 29.8, 29.8, 29.7, 29.6, 29.5, 29.4, 29.0, 26.9, 26.5, 22.8, 14.3 ppm; IR (neat): *ṽ*=2916 (vs), 2851 (vs), 1684 (s), 1605 (m), 1469 (m), 1355 (m), 1264 (m), 1256 cm^−1^ (m); MS (70 eV): *m*/*z* (%): 487 (7) [*M*]^**+**.^, 459 (5) [*M*‐N_2_]^**+**.^, 235 (100) [*M−C*_16_H_32_N_2_]^**+**.^, 234 (39) [*M−C*_16_H_33_N_2_]^+^; HRMS (LC–IT‐TOF, elution with 0.1 % (*v*/*v*) formic acid in methanol) *m*/*z*: [*M*+H]^+^ Calcd for C_32_H_46_N_3_O 488.3635; Found 488.3627, mass error=1.64 ppm, C (32 : 12).

### General procedure for the synthesis of compounds 8 a–g under IR irradiation

A Carius tube (ø=1.6 cm) with a screw cap and equipped with a magnetic stirrer was charged with pentafluorobenzene **7** (0.5 or 1.0 mmol), aryl iodide (0.5 or 1.0 mmol), Pd/AlO(OH) nanoparticles (0.15 mol%), PPh_3_ (3 mol%), Ag_2_CO_3_ (1.0 mmol). The resulting heterogeneous reaction mixture was heated under an IR lamp (the distance between the bottom of Carius tube and the bulb was set to 7 cm, Figure 2). After 15 min, the mixture was cooled to room temperature and the crude product was purified by column chromatography on silica gel.

**2,3,4,5,6‐Pentafluoro‐1,1′‐biphenyl (8** **a)**:[^17b^, ^19^] Compound **8** **a** was synthesized from **7** (168 mg, 1.0 mmol) and iodobenzene (204 mg, 1.0 mmol) in accordance with the general procedure. Purification by column chromatography (*n*‐hexane) afforded 212 mg of compound **8** **a** as a white solid (87 % yield). Analytical data agree with those previously reported in the literature.[^17b^, ^19^] *R*_f_ (*n*‐hexane)=0.59; ^1^H NMR (500 MHz, CDCl_3_): *δ*=7.52–7.45 (m, 3H), 7.44–7.40 ppm (m, 2H); ^13^C NMR (126 MHz, CDCl_3_): *δ*=144.3 (m, incl. app. d, *J*=247.7 Hz), 140.6 (m, incl. app. d, *J*=253.7 Hz), 138.0 (m, incl. app. d, *J*=252.9 Hz), 130.3, 129.4, 128.9, 126.6, 116.1 ppm (td, *J*=17.3, 3.9 Hz); MS (70 eV): *m*/*z* (%): 244 (100) [*M*]^**+**.^, 243 (8) [*M*‐H]^+^, 225 (18) [*M*‐F]^+^.

**2,3,4,4′,5,6‐Hexafluoro‐1,1′‐biphenyl (8** **b)**:[^17b^, ^20^] Compound **8** **b** was synthesized from **7** (168 mg, 1.0 mmol) and 1‐fluoro‐4‐iodobenzene (222 mg, 1.0 mmol) in accordance with the general procedure. Purification by column chromatography (*n*‐hexane) afforded 199 mg of compound **8** **b** as a white solid (76 % yield). Analytical data are in agreement with those previously reported in the literature.[^17b^, ^20^] *R*_f_ (*n*‐hexane)=0.62; ^1^H NMR (500 MHz, CDCl_3_): *δ*=7.43–7.39 (m, 2H), 7.19 ppm (t like, *J*=8.7 Hz, 2H); ^13^C NMR (126 MHz, CDCl_3_): *δ*=163.4 (d, *J*=249.9 Hz), 144.3 (m, incl. app. d, *J*=247.5 Hz), 140.6 (m, incl. app. d, *J*=254.2 Hz), 138.1, (m, incl. app. d, *J*=253.2 Hz), 132.2 (d, *J*=8.5 Hz), 122.4, 116.1 (d, *J*=22.0 Hz), 115.0 ppm (td, *J*=17.0, 3.8 Hz); MS (70 eV): *m*/*z* (%): 262 (100) [*M*]^**+**.^, 243 (19) [*M*‐F]^+^, 242 (22) [*M*‐HF]^**+**.^.

**2,3,4,5,6‐Pentafluoro‐2′‐methoxy‐1,1′‐biphenyl (8** **c)**:[^17f^, ^21^] Compound **8** **c** was synthesized from **7** (168 mg, 1.0 mmol) and 1‐iodo‐2‐methoxybenzene (234 mg, 1.0 mmol) in accordance with the general procedure. Purification by column chromatography (*n*‐hexane) afforded 149 mg of compound **8** **c** as a white solid (54 % yield). Analytical data are in agreement with those previously reported in the literature.[^17f^, ^21^] *R*_f_ (*n*‐hexane)=0.35; ^1^H NMR (500 MHz, CDCl_3_): *δ*=7.47 (ddd, *J*=8.3, 7.5, 1.7 Hz, 1H), 7.23 (d br, *J*=7.5 Hz, 1H), 7.06 (td, *J*=7.5, 1.0 Hz, 1H), 7.03 (d, *J*=8.3 Hz, 1H), 3.81 ppm (s, 3H); ^13^C NMR (126 MHz, CDCl_3_): *δ*=157.3, 144.6 (m, incl. app. d, *J*=247.1 Hz), 140.7 (m, incl. app. d, *J*=252.7 Hz), 137.7 (d, *J*=252.0 Hz), 131.9, 131.3, 120.8, 115.4, 113.0 (td, *J*=19.2, 3.9 Hz), 111.4, 55.8 ppm; MS (70 eV): *m*/*z* (%): 274 (100) [*M*]^**+**.^, 259 (25) [*M−C*H_3_]^+^.

**2,3,4,5,6‐Pentafluoro‐3′‐methyl‐1,1′‐biphenyl (8** **d)**:[^20^, ^22^] Compound **8** **d** was synthesized from **7** (168 mg, 1.0 mmol) and 3‐iodotoluene (218 mg, 1.0 mmol) in accordance with the general procedure. Purification by column chromatography (*n*‐hexane) afforded 200 mg of compound **8** **d** as a white solid (78 % yield). Analytical data are in agreement with those previously reported in the literature.[^20^, ^22^] *R*_f_ (*n*‐hexane)=0.61; ^1^H NMR (500 MHz, CDCl_3_): *δ*=7.38 (t, *J*=7.6 Hz, 1H), 7.28 (d, *J*=7.6 Hz, 1H), 7.24–7.19 (m, 2H), 2.42 ppm (s, 3H); ^13^C NMR (126 MHz, CDCl_3_): *δ*=144.3 (m, incl. app. d, *J*=247.4 Hz), 140.5 (m, incl. app. d, *J*=253.5 Hz), 138.7, 138.0 (m, incl. app. d, *J*=250.6 Hz), 130.9, 130.2, 128.7, 127.3, 126.4, 116.3 (td, *J*=17.2, 3.9 Hz), 21.5 ppm; MS (70 eV): *m*/*z* (%): 258 (100) [*M*]^**+**.^, 257 (25) [*M*‐H]^+^, 239 (15) [*M*‐F]^+^.

**2,3,4,5,6‐Pentafluoro‐4′‐(trifluoromethyl)‐1,1′‐biphenyl (8** **e)**:^[23]^ Compound **8** **e** was synthesized from **7** (168 mg, 1.0 mmol) and 1‐iodo‐4‐(trifluoromethyl)benzene (272 mg, 1.0 mmol) in accordance with the general procedure. Purification by column chromatography (*n*‐hexane) afforded 241 mg of compound **8** **e** as a white solid (77 % yield). *R*_f_ (*n*‐hexane)=0.52; ^1^H NMR (500 MHz, CDCl_3_): *δ*=7.76 (d, *J*=8.1 Hz, 2H), 7.56 ppm (d, *J*=8.1 Hz, 2H); ^13^C NMR (126 MHz, CDCl_3_): *δ*=144.3 (m, incl. app. d, *J*=249.1 Hz), 141.2 (m, incl. app. d, *J*=255.4 Hz), 138.1 (m, incl. app. d, *J*=253.4 Hz), 131.7 (q, *J*=32.8 Hz), 130.8, 130.3, 125.9 (q, *J*=3.7 Hz), 123.9 (q, *J*=272.3 Hz), 114.7 ppm (td, *J*=17.0, 4.0 Hz); MS (70 eV): *m*/*z* (%): 312 (100) [*M*]^**+**.^, 293 (27) [*M*‐F]^+^, 243 (19) [*M−C*F_3_]^+^.

**2,3,4,5,6‐pentafluoro‐3′,5′‐dimethyl‐1,1′‐biphenyl (8** **f)**:[^20^, ^22^] Compound **8** **f** was synthesized from **7** (84 mg, 0.5 mmol) and 1‐iodo‐3,5‐dimethylbenzene (116 mg, 0.5 mmol) in accordance with the general procedure. Purification by column chromatography (*n*‐hexane) afforded 117 mg of compound **8** **f** as a white solid (86 % yield). Analytical data agree with those previously reported in the literature.[^20^, ^22^] *R*_f_ (*n*‐hexane)=0.66; ^1^H NMR (500 MHz, CDCl_3_): *δ*=7.10 (s br, 1H), 7.02 (s br, 2H), 2.38 ppm (s, 6H); ^13^C NMR (126 MHz, CDCl_3_): *δ*=144.3 (m, incl. app. d, *J*=247.0 Hz), 140.4 (m, incl. app. d, *J*=253.3 Hz), 138.5, 137.9 (m, incl. app. d, *J*=252.7 Hz), 131.1, 128.0, 126.3, 116.4 (td, *J*=18.1, 4.2 Hz), 21.4 ppm; MS (70 eV): *m*/*z* (%): 272 (100) [*M*]^**+**.^, 271 (10) [*M*‐H]^+^, 257 (75) [*M−C*H_3_]^+^.

**Methyl 2′,3′,4′,5′,6′‐pentafluoro‐[1,1′‐biphenyl]‐4‐carboxylate (8** **g)**:^[24]^ Compound **8** **g** was synthesized from **7** (168 mg, 1.0 mmol) and methyl 4‐iodobenzoate (262 mg, 1.0 mmol) in accordance with the general procedure. Purification by column chromatography (*n*‐hexane/ethyl acetate=9.5:0.5) afforded 253 mg of compound **8** **g** as a white solid (84 % yield). Analytical data are in agreement with those previously reported in the literature.^[24]^ *R*_f_ (*n*‐hexane/ethyl acetate=9.5:0.5)=0.24; ^1^H NMR (500 MHz, CDCl_3_): *δ*=8.16 (d, *J*=8.5 Hz, 2H), 7.51 (d, *J*=8.5 Hz, 2H), 3.96 ppm (s, 3H); ^13^C NMR (126 MHz, CDCl_3_): *δ*=166.5, 144.2 (m, incl. app. d, *J*=248.8 Hz), 141.0 (m, incl. app. d, *J*=255.0 Hz), 138.1 (m, incl. app. d, *J*=253.4 Hz), 131.1, 130.4, 130.0, 115.1 (td, *J*=17.0, 3.9 Hz), 52.4 ppm; MS (70 eV): *m*/*z* (%): 302 (43) [*M*]^**+**.^, 271 (100) [*M*‐OCH_3_]^+^, 243 (23) [*M−C*O_2_CH_3_]^+^.

**1‐(2′,3′,4′,5′,6′‐pentafluoro‐[1,1′‐biphenyl]‐4‐yl)ethanone (8** **h)**:[^18^, ^24^] A Carius tube (ø=1.6 cm) with a screw cap and equipped with a magnetic stirrer was charged with **7** (168 mg, 1.0 mmol), 1‐(4‐iodophenyl)ethanone (246 mg, 1.0 mmol), Pd/AlO(OH) nanoparticles (32 mg), PPh_3_ (8 mg), Ag_2_CO_3_ (276 mg, 1.0 mmol). The resulting heterogeneous reaction mixture was heated under an IR lamp (the distance between the bottom of Carius tube and the bulb was set to 7 cm, Figure 1). After 15 min, the mixture was cooled to room temperature, then quenched with a saturated aqueous solution of NH_4_Cl (20 mL) and extracted with dichloromethane (3×30 mL). The combined organic extracts were washed with an aqueous solution of NaCl (3×20 mL), dried with Na_2_SO_4_, and concentrated under vacuum. The crude product was purified by column chromatography (*n*‐hexane/ethyl acetate=90 : 5) afforded 195 mg of compound **8** **g** as a yellow solid (68 % yield). Analytical data are in agreement with those previously reported in the literature.[^18^, ^24^] *R*_f_ (*n‐*hexane/ethyl acetate=90 : 5)=0.21; ^1^H NMR (500 MHz, CDCl_3_): *δ*=8.08 (d, *J*=8.5 Hz, 2H), 7.54 (d, *J*=8.5 Hz, 2H), 2.66 ppm (s, 3H); ^13^C NMR (126 MHz, CDCl_3_): *δ*=197.2, 144.1 (m, incl. app. d, *J*=248.9 Hz), 140.8 (m, incl. app. d, *J*=255.2 Hz), 137.9 (m, incl. app. d, *J*=253.6 Hz), 137.5, 131.0, 130.5, 128.5, 114.8 (td, *J*=17.0, 4.1 Hz), 26.6 ppm; MS (70 eV): *m*/*z* (%): 286 (9) [*M*]^**+**.^, 271 (100) [*M−C*H_3_]^+^, 243 (35) [*M−C*(O)CH_3_]^+^.

## Conflict of interest

The authors declare no conflict of interest.

## Supporting information

As a service to our authors and readers, this journal provides supporting information supplied by the authors. Such materials are peer reviewed and may be re‐organized for online delivery, but are not copy‐edited or typeset. Technical support issues arising from supporting information (other than missing files) should be addressed to the authors.

Supporting Information
