# Supplementary material for: Infrared Irradiation‐Assisted Solvent‐Free Pd‐Catalyzed (Hetero)aryl‐aryl Coupling via C−H Bond Activation
Source: ChemSusChem. 2021 Jul 22;14(16):3391–401. doi: 10.1002/cssc.202101070 (PMC8456959; doi:10.1002/cssc.202101070)
Supplement: Supplementary file 1 — Supporting Information [file CSSC-14-3391-s001.pdf]

ChemSusChem

Supporting Information

Infrared Irradiation-Assisted Solvent-Free Pd-Catalyzed (Hetero)aryl-aryl Coupling via C–H Bond Activation

Gianluigi Albano[+], Gianfranco Decandia[+], Maria Annunziata M. Capozzi, Nicola Zappimbulso, Angela Punzi,* and Gianluca M. Farinola*© 2021 The Authors. ChemSusChem published by Wiley-VCH GmbH. This is an open access article under the terms of the Creative Commons Attribution License, which permits use, distribution and reproduction in any medium, provided the original work is properly cited.

Table of Contents

NMR Spectra of compounds 2a-f 3-8

NMR Spectra of compounds 4a-f 9-14

NMR Spectra of compounds 6a-f 15-20

NMR Spectra of compounds 8a-g 21-28

HRMS Spectra of compounds 6e and 6f...29-30

NMR Spectra

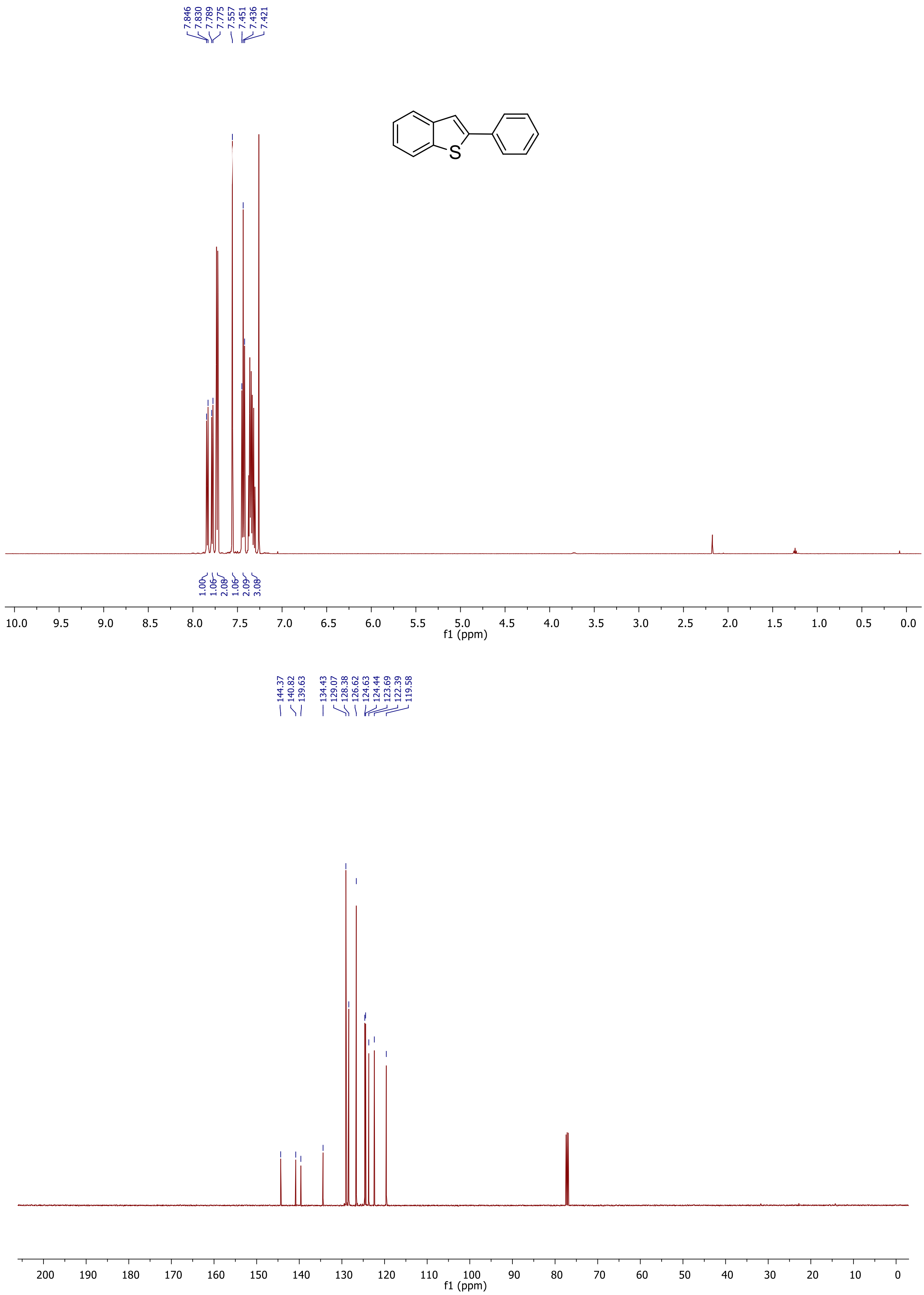

Figure S1. ^{1}H NMR and ^{13}C NMR spectra of compound **2a** (500 and 126 MHz, $CDCl_3$).

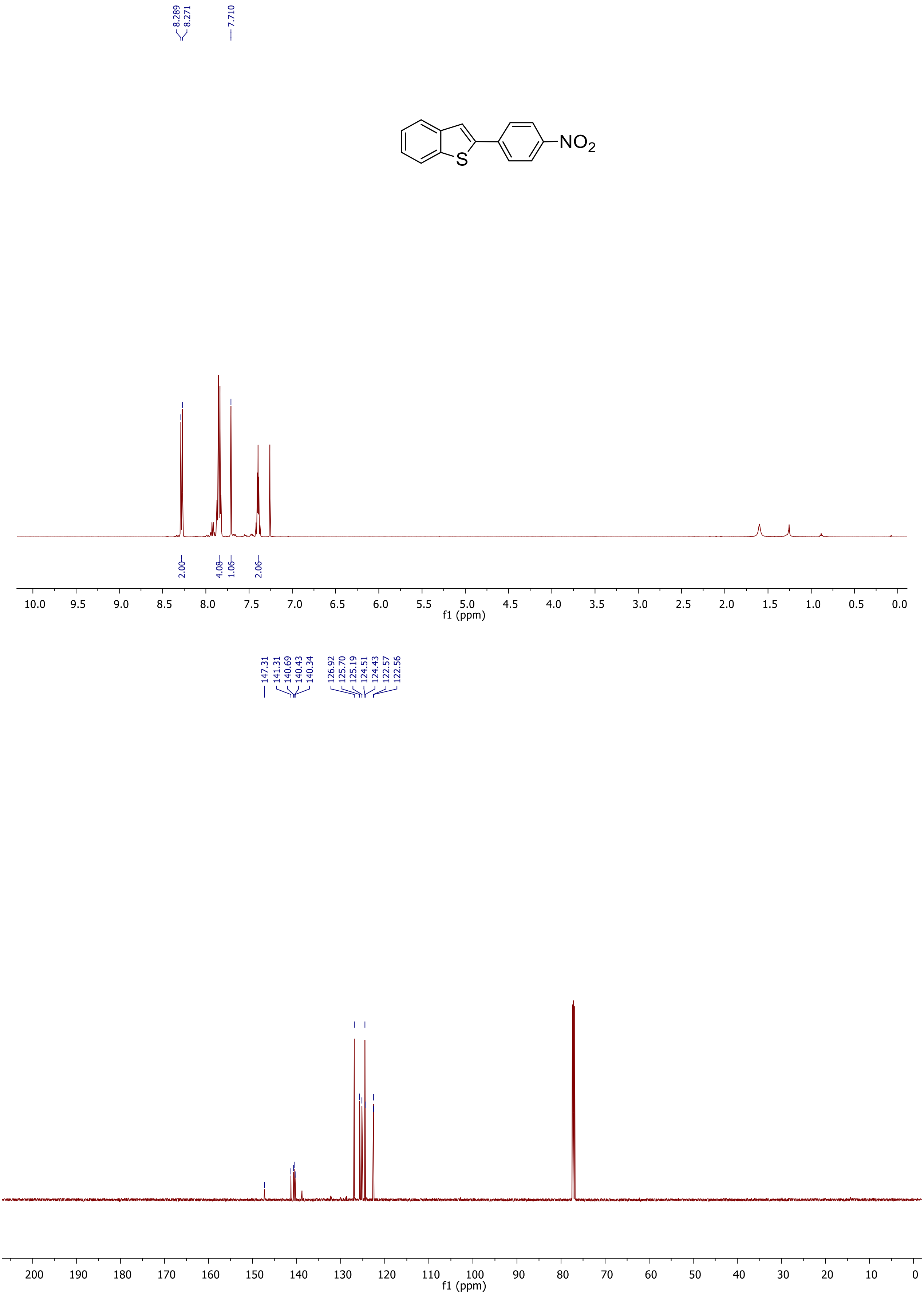

Figure S2. ^{1}H NMR and ^{13}C NMR spectra of compound **2b** (500 and 126 MHz, $CDCl_3$).

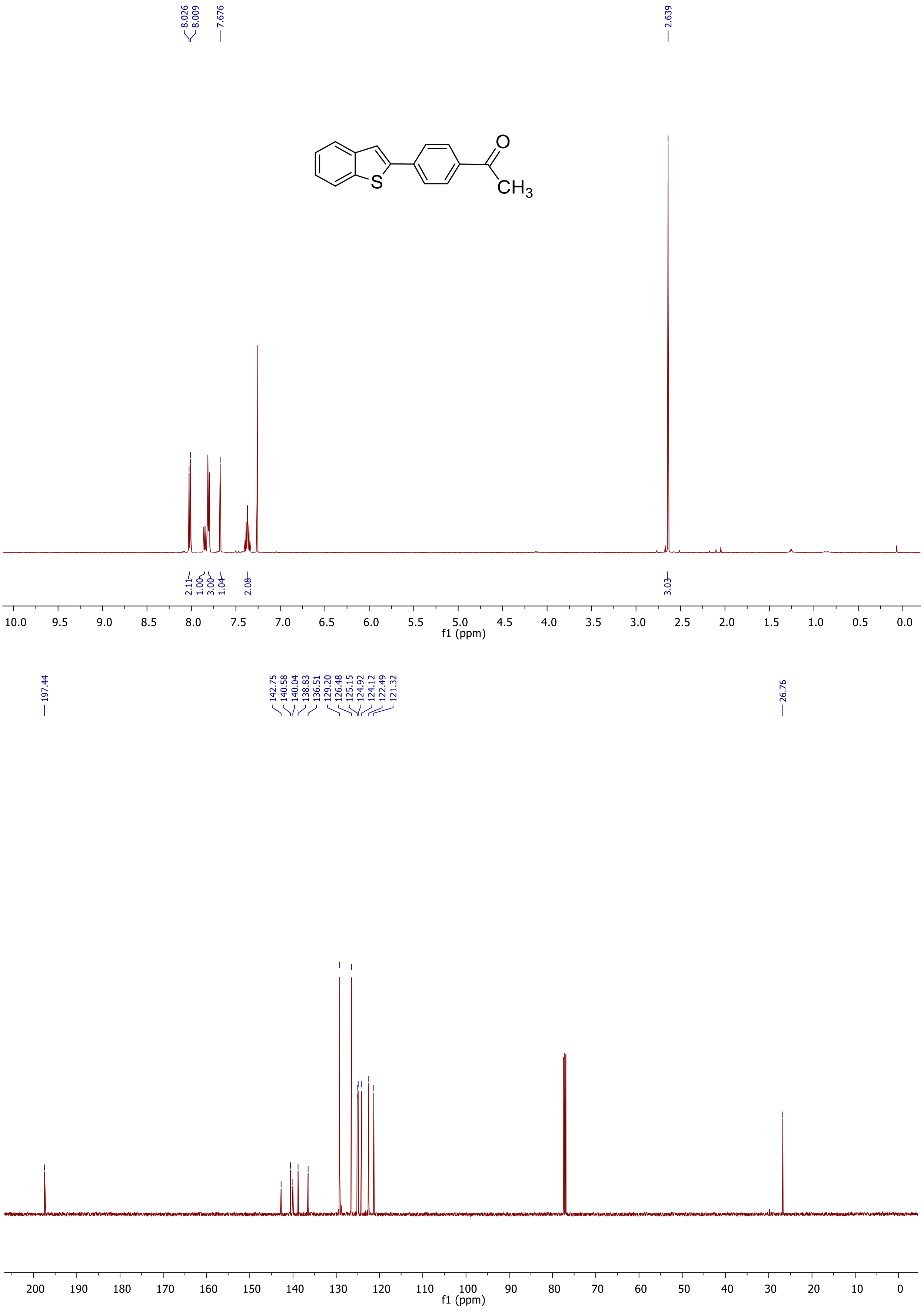

Figure S3. ^{1}H NMR and ^{13}C NMR spectra of compound **2c** (500 and 126 MHz, $CDCl_3$).

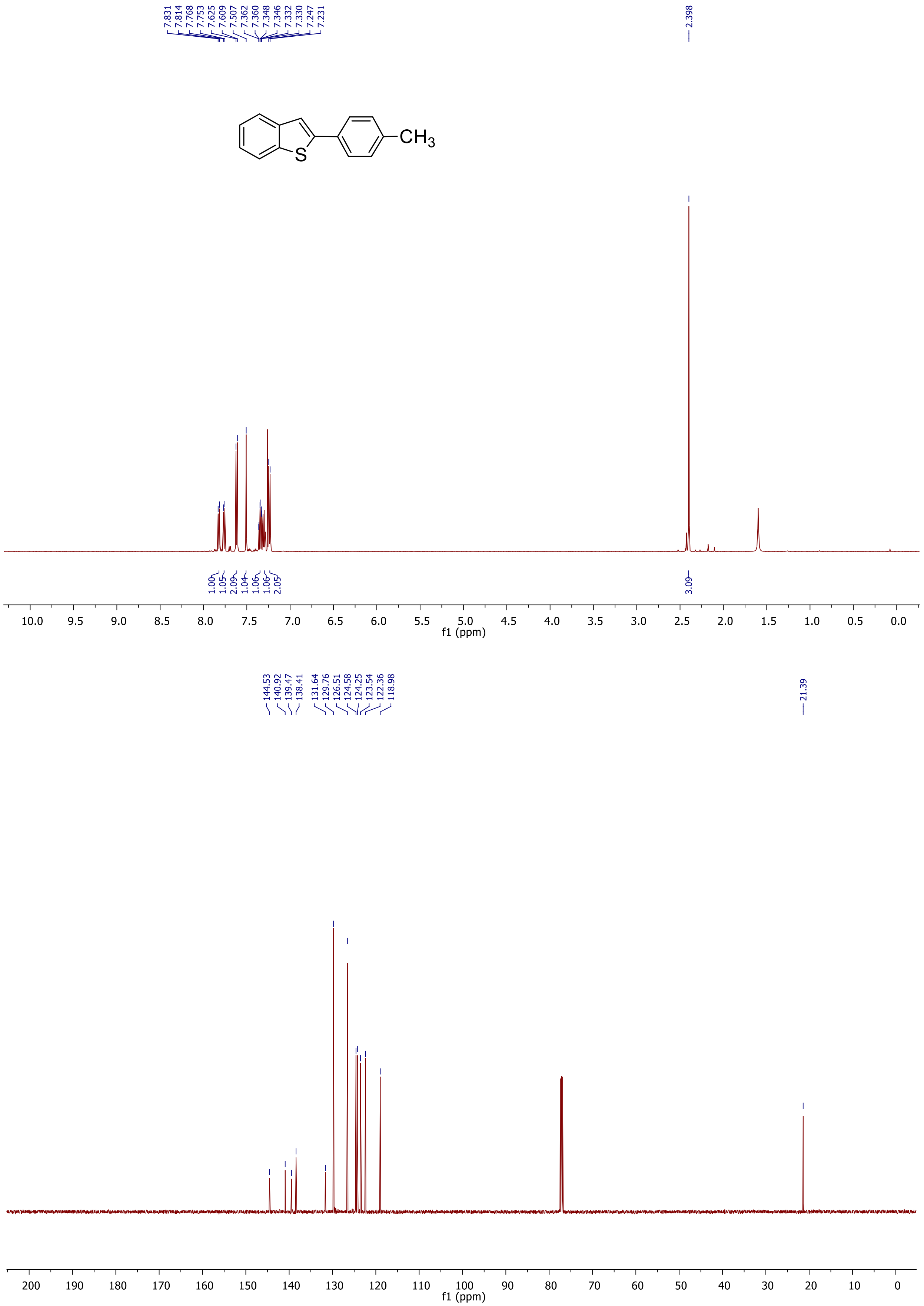

Figure S4. ^{1}H NMR and ^{13}C NMR spectra of compound **2d** (500 and 126 MHz, $CDCl_3$).

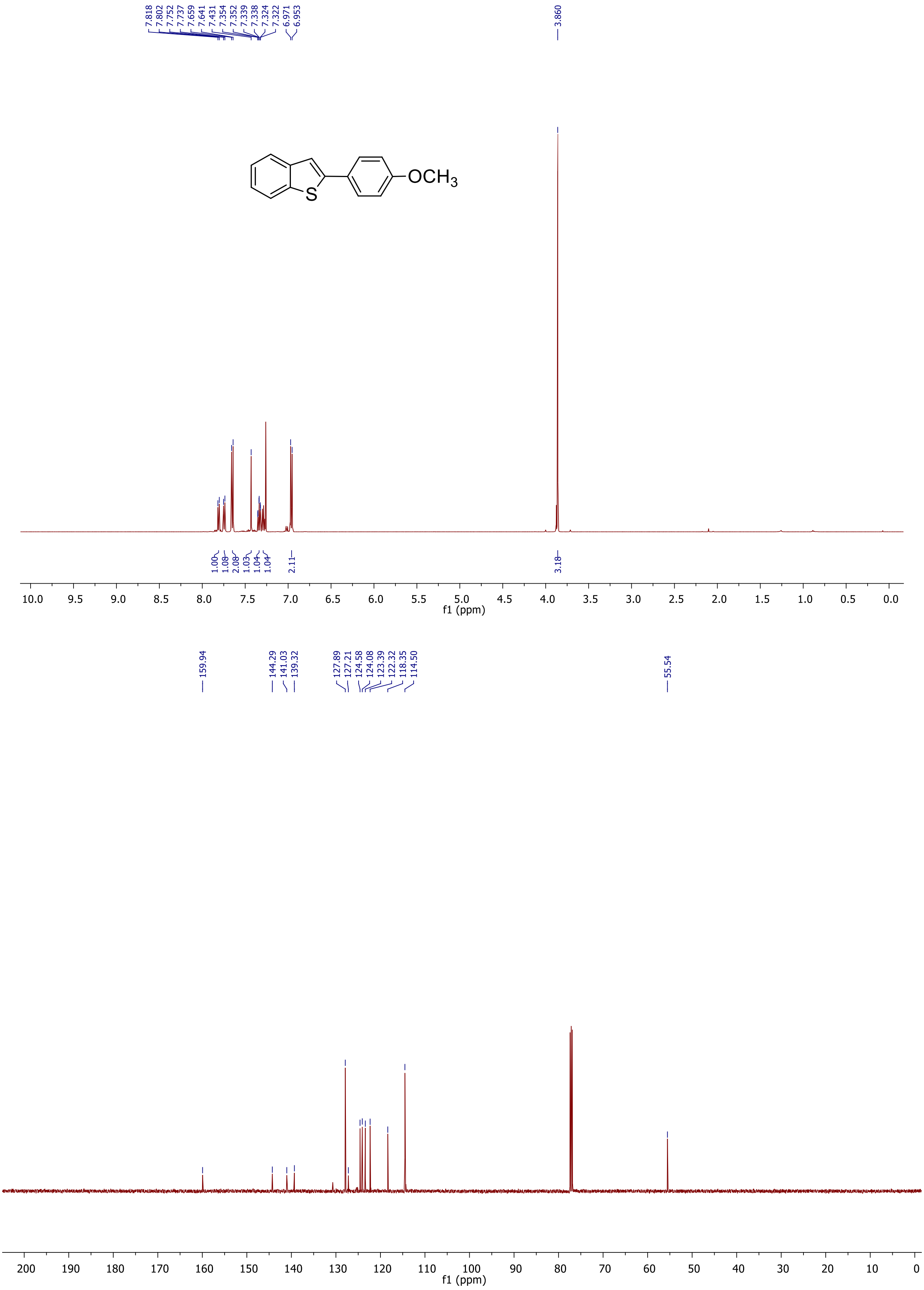

Figure S5. ^{1}H NMR and ^{13}C NMR spectra of compound **2e** (500 and 126 MHz, $CDCl_3$).

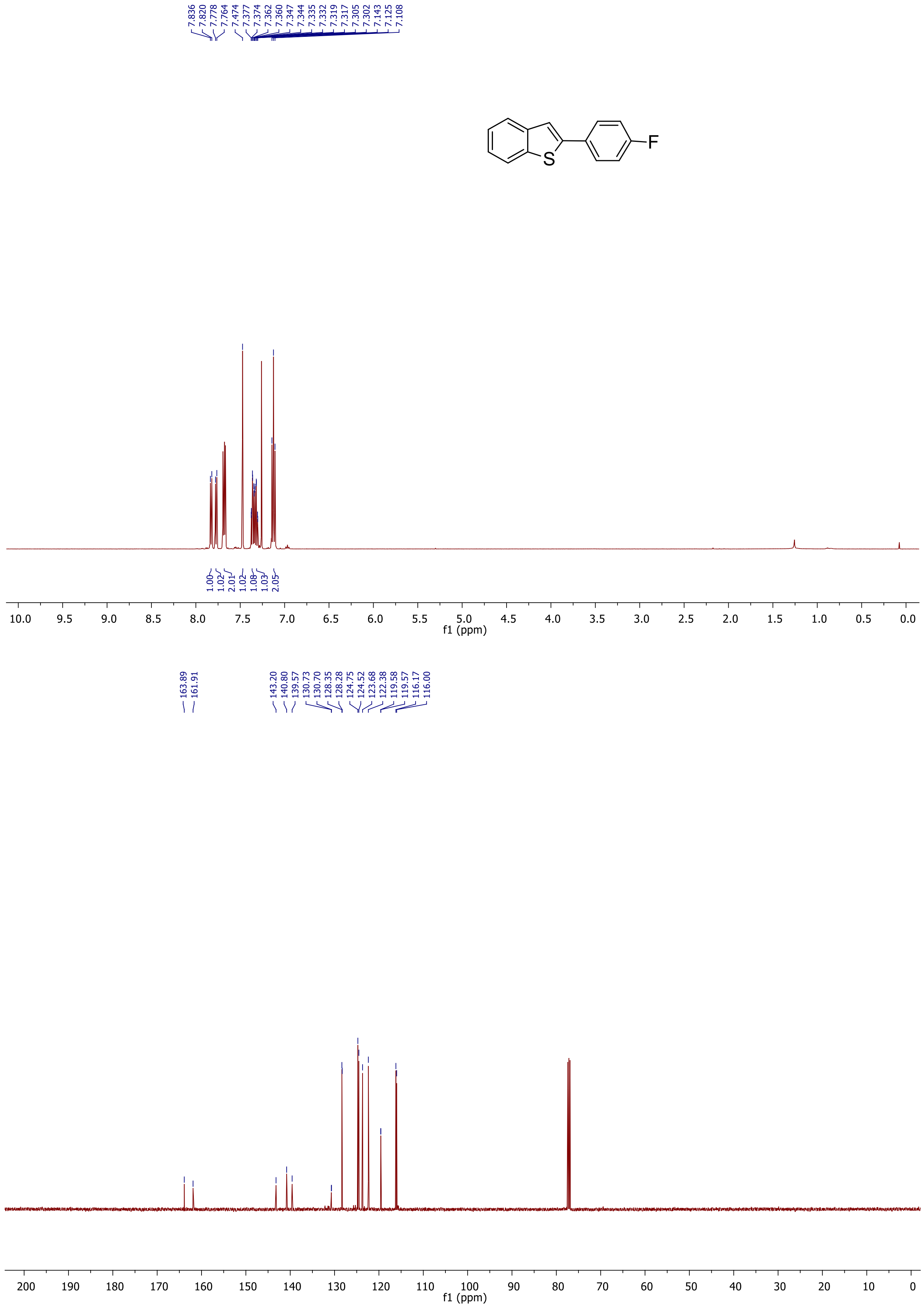

Figure S6. ^{1}H NMR and ^{13}C NMR spectra of compound **2f** (500 and 126 MHz, $CDCl_3$).

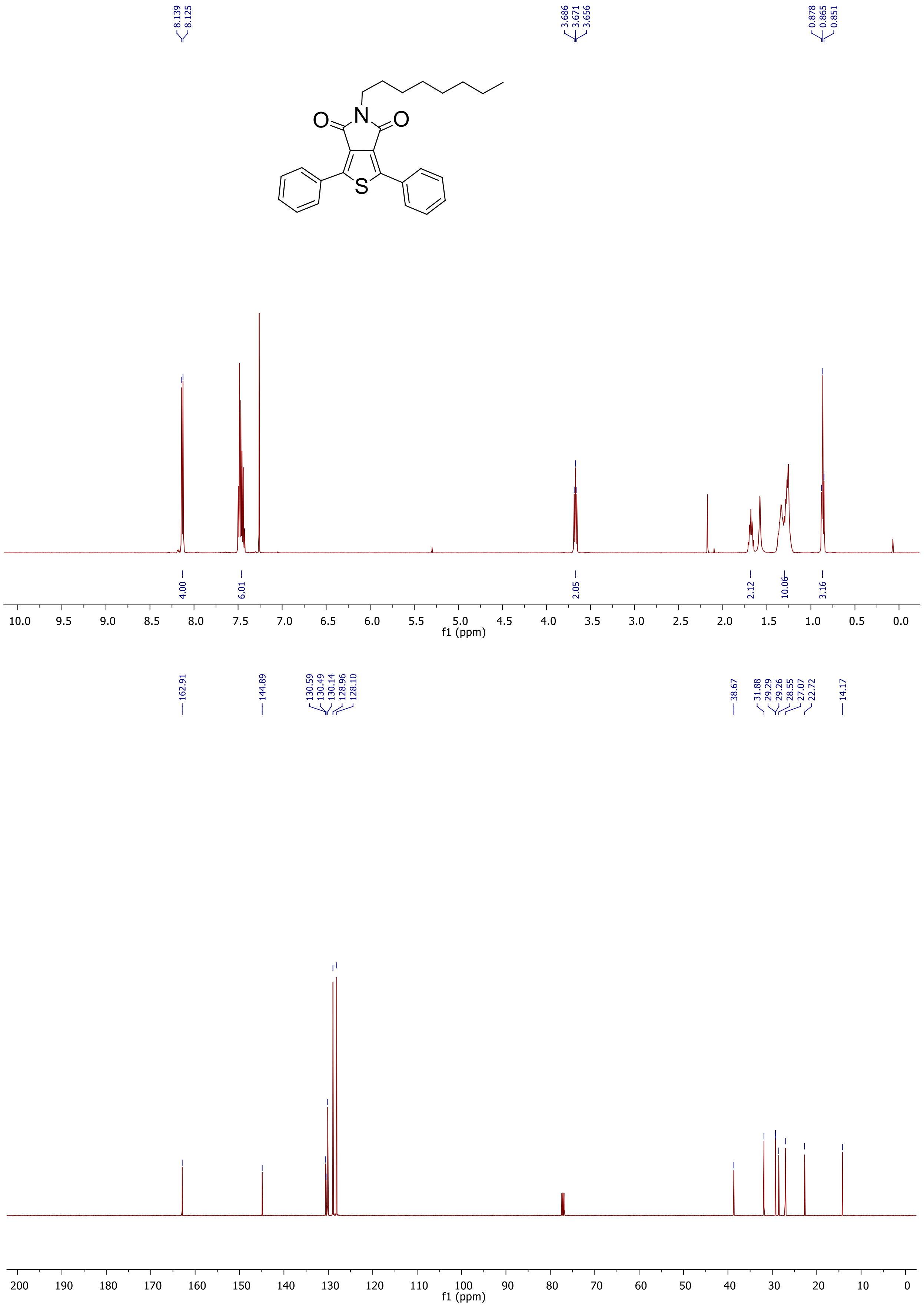

Figure S7. ^{1}H NMR and ^{13}C NMR spectra of compound **4a** (500 and 126 MHz, $CDCl_3$).

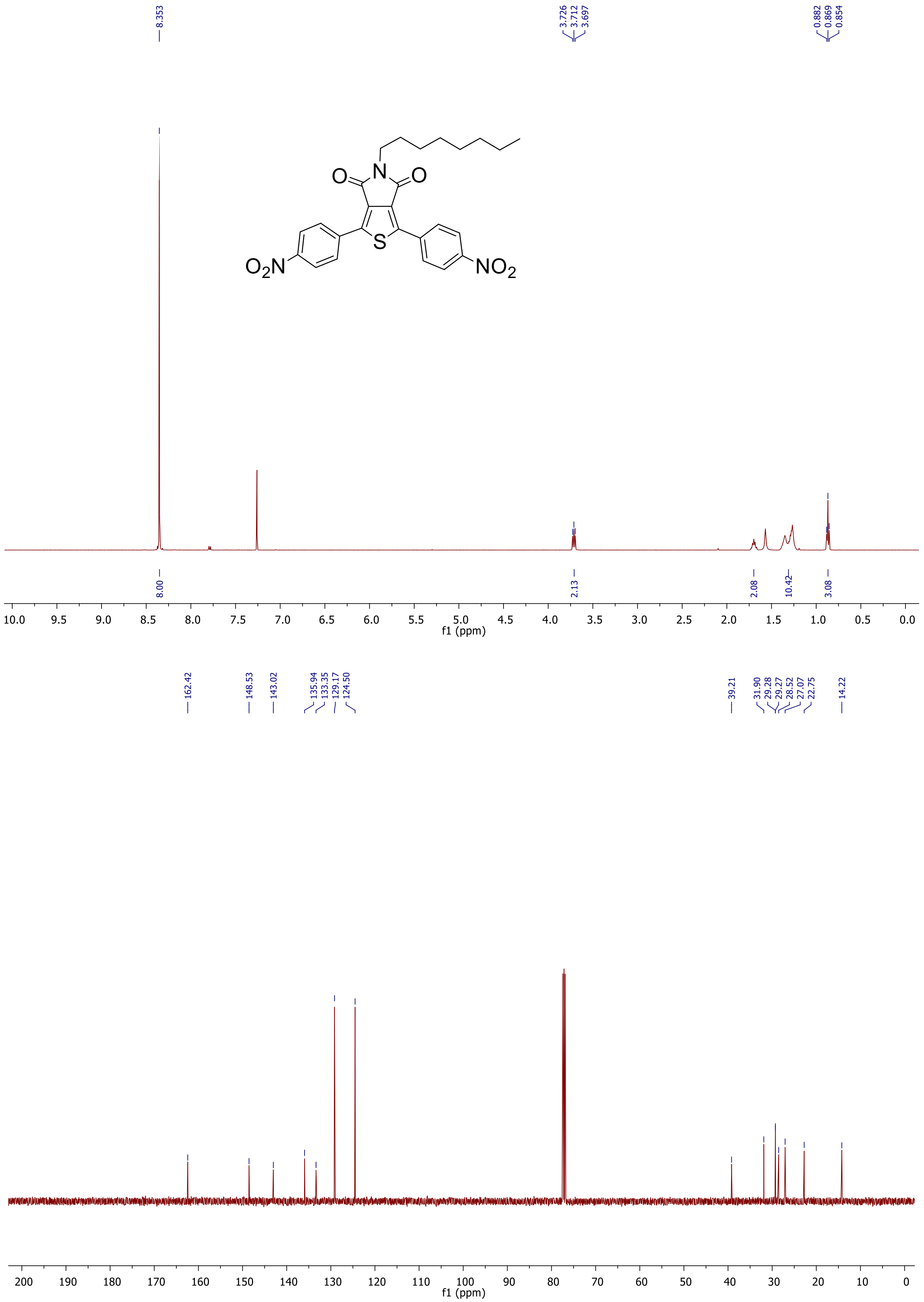

Figure S8. ^{1}H NMR and ^{13}C NMR spectra of compound **4b** (500 and 126 MHz, $CDCl_3$).

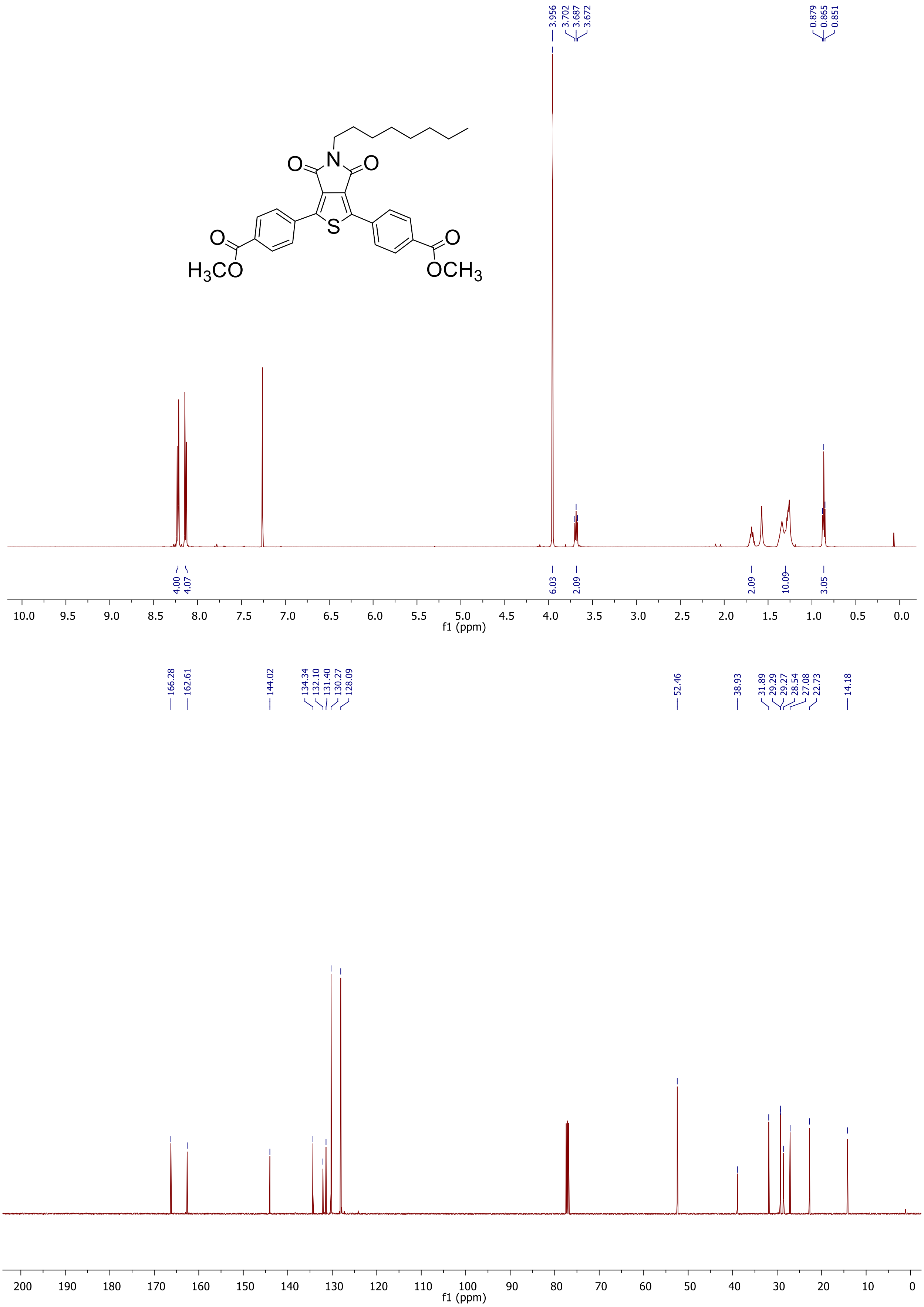

Figure S9. ^{1}H NMR and ^{13}C NMR spectra of compound **4c** (500 and 126 MHz, $CDCl_3$).

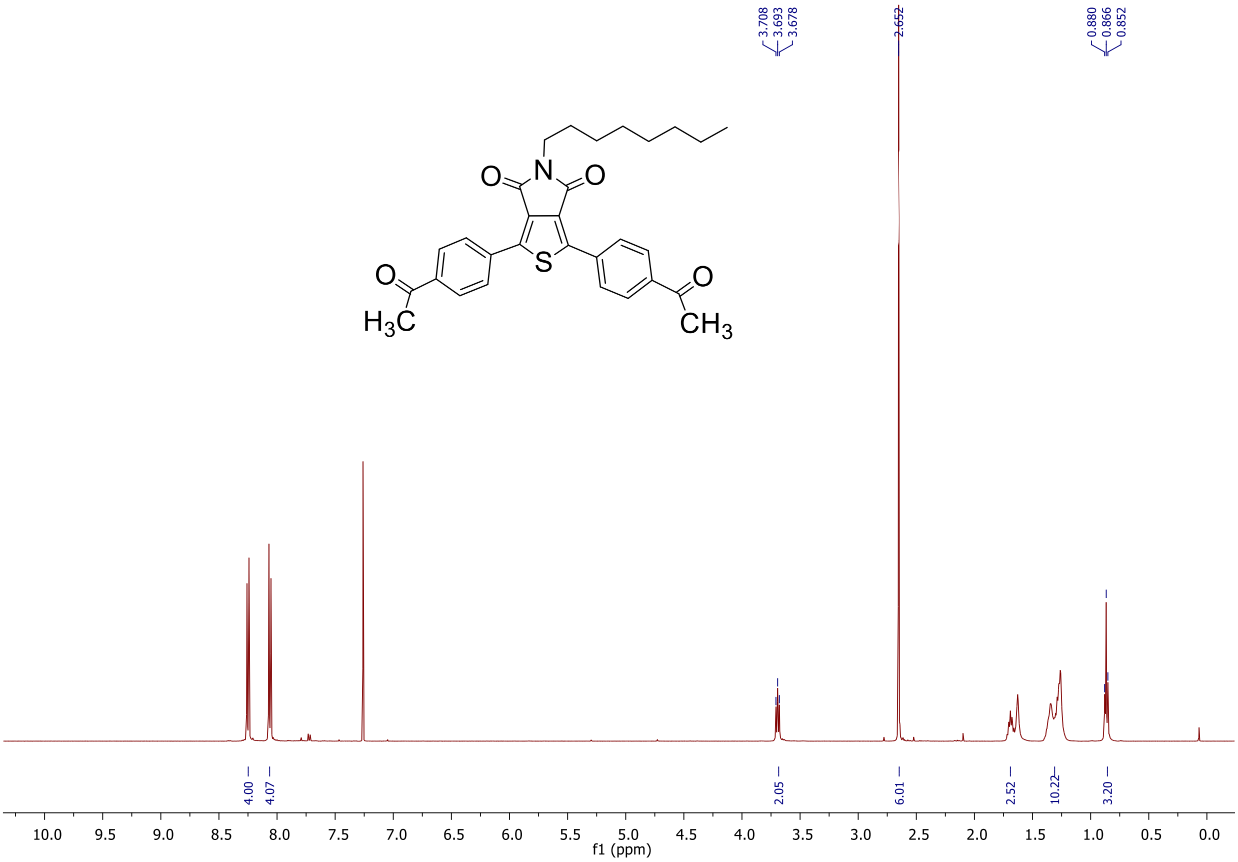

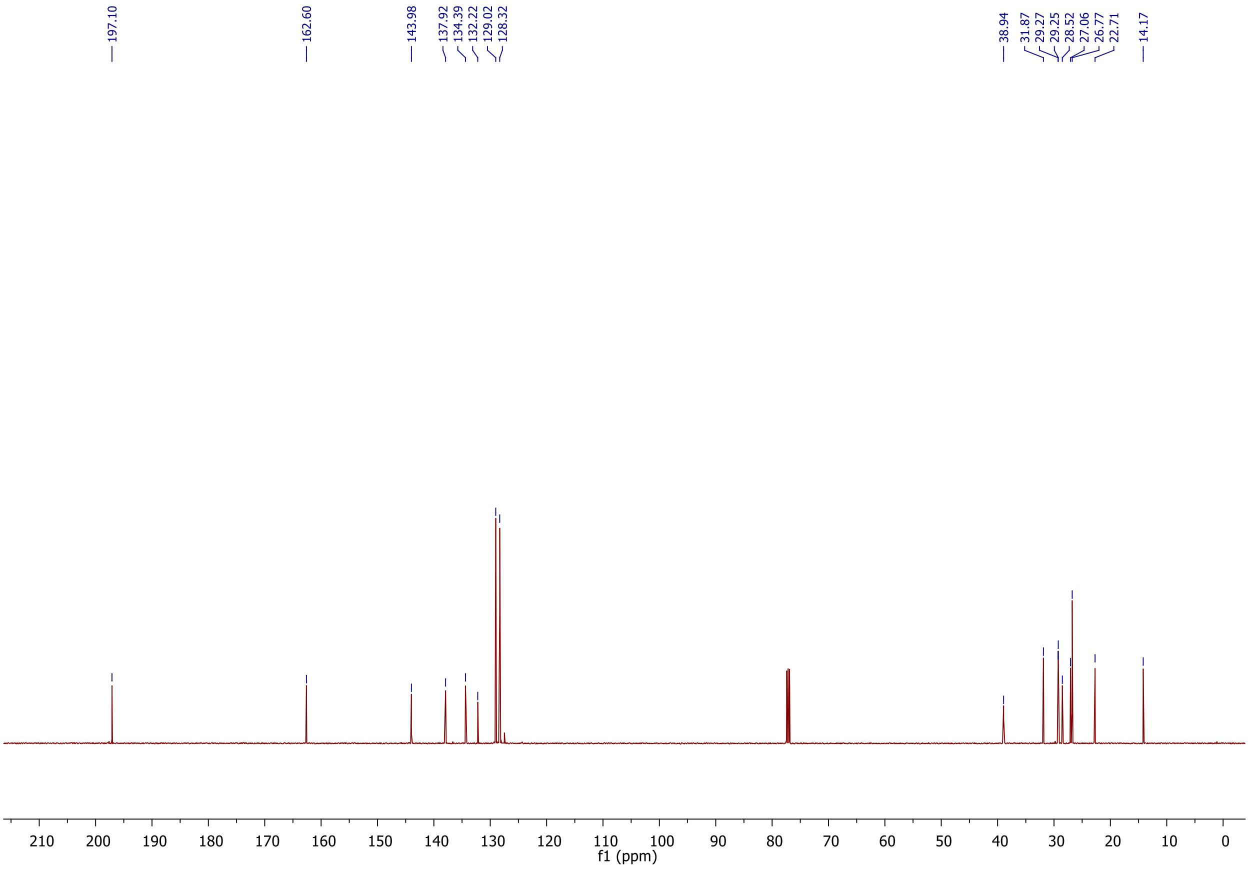

Figure S10. ^{1}H NMR and ^{13}C NMR spectra of compound **4d** (500 and 126 MHz, $CDCl_3$).

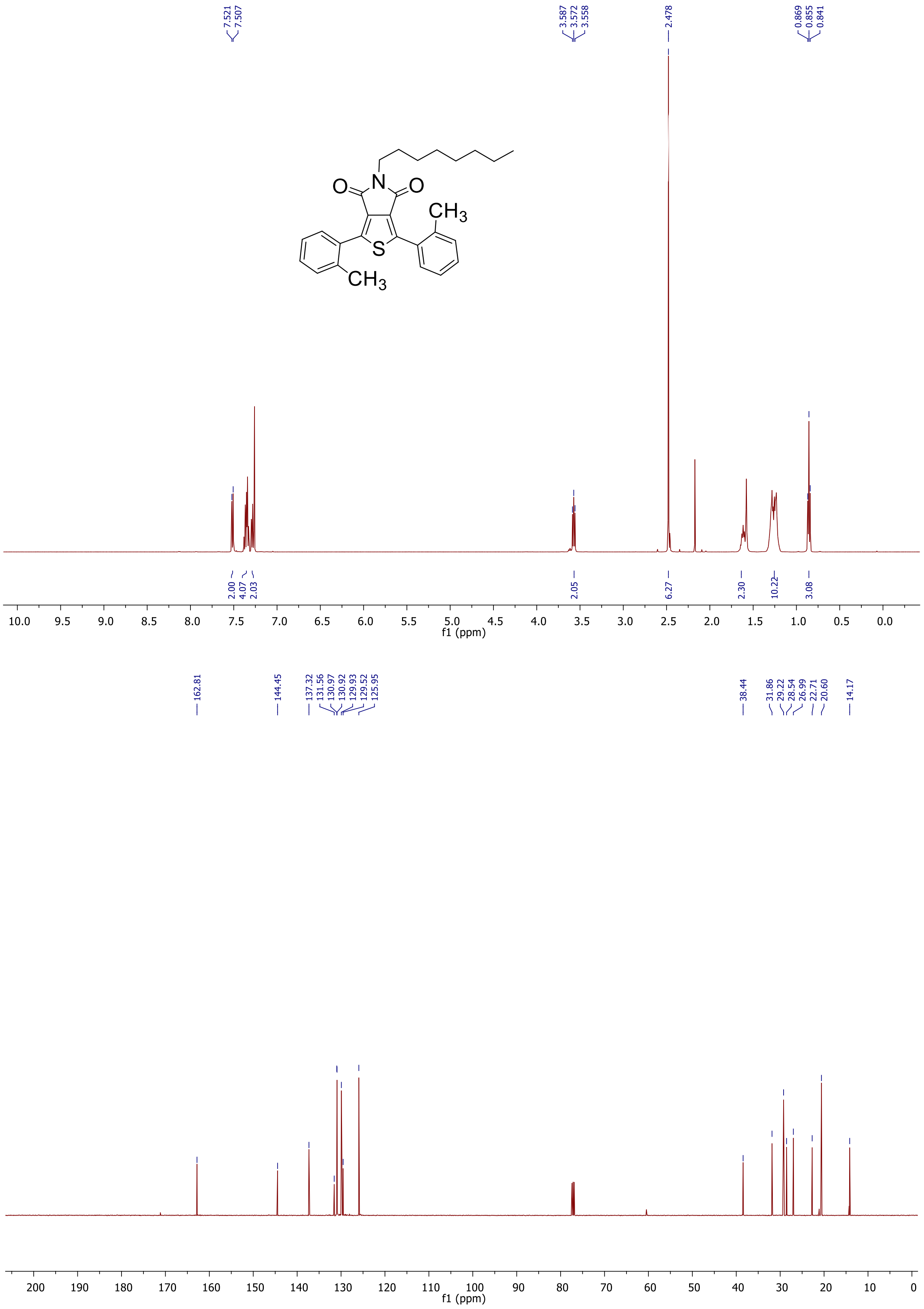

Figure S11. ^{1}H NMR and ^{13}C NMR spectra of compound **4e** (500 and 126 MHz, $CDCl_3$).

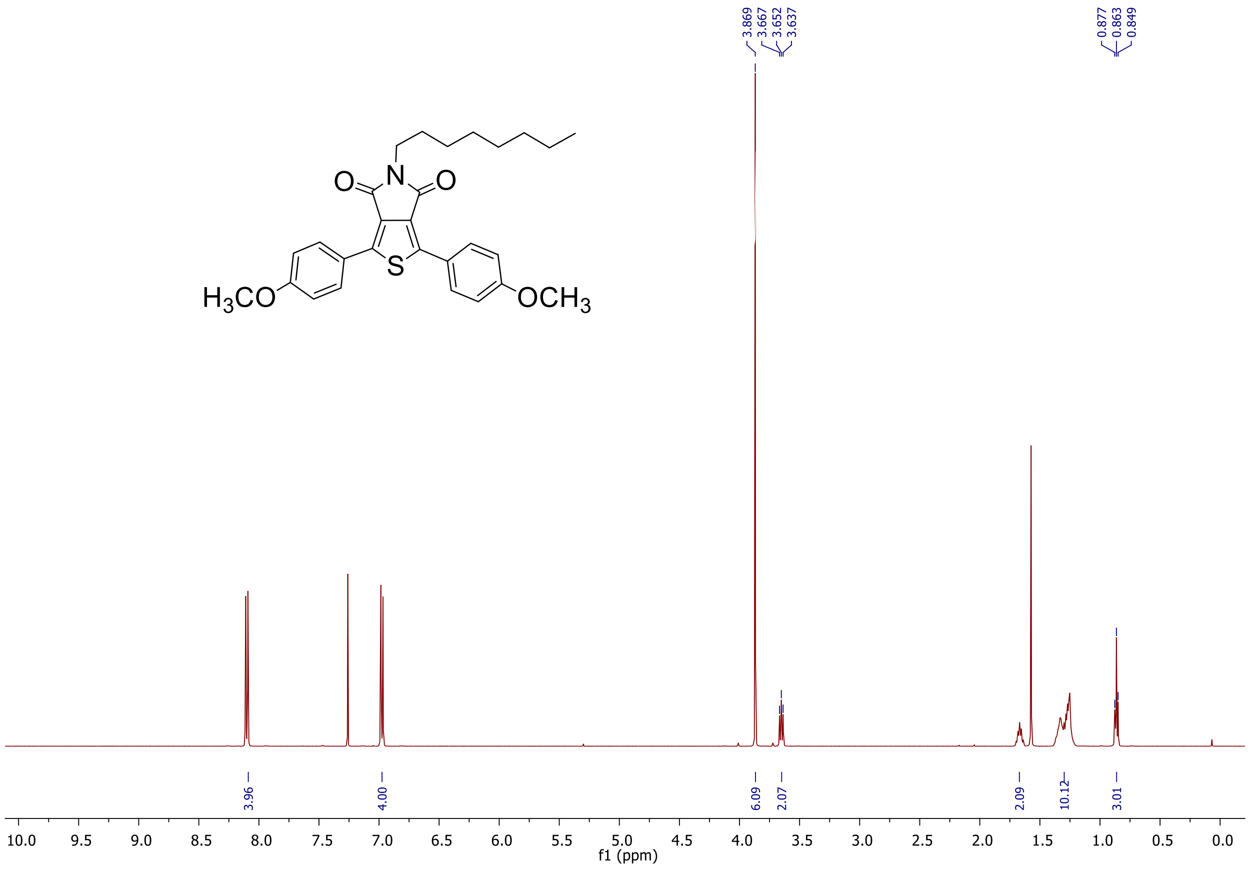

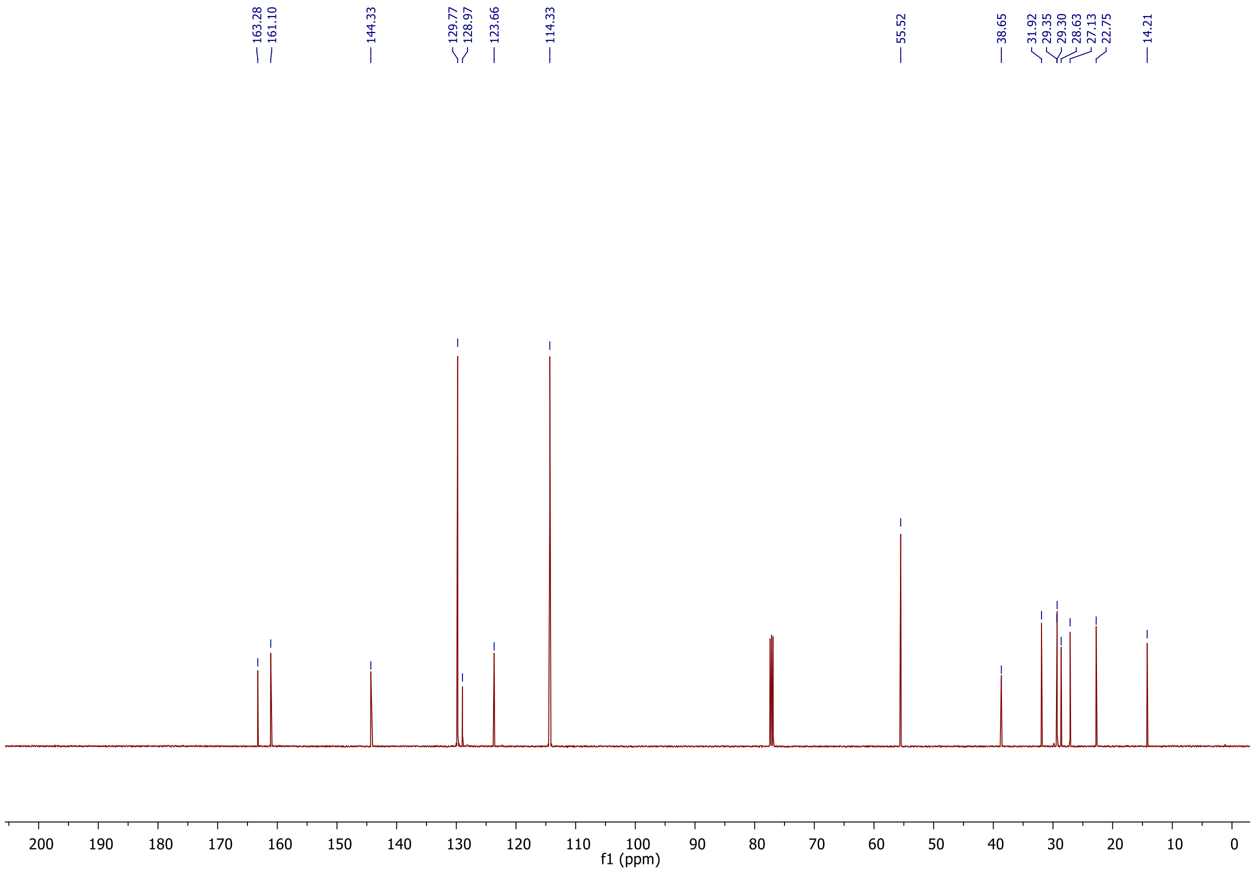

Figure S12. ^{1}H NMR and ^{13}C NMR spectra of compound **4f** (500 and 126 MHz, $CDCl_3$).

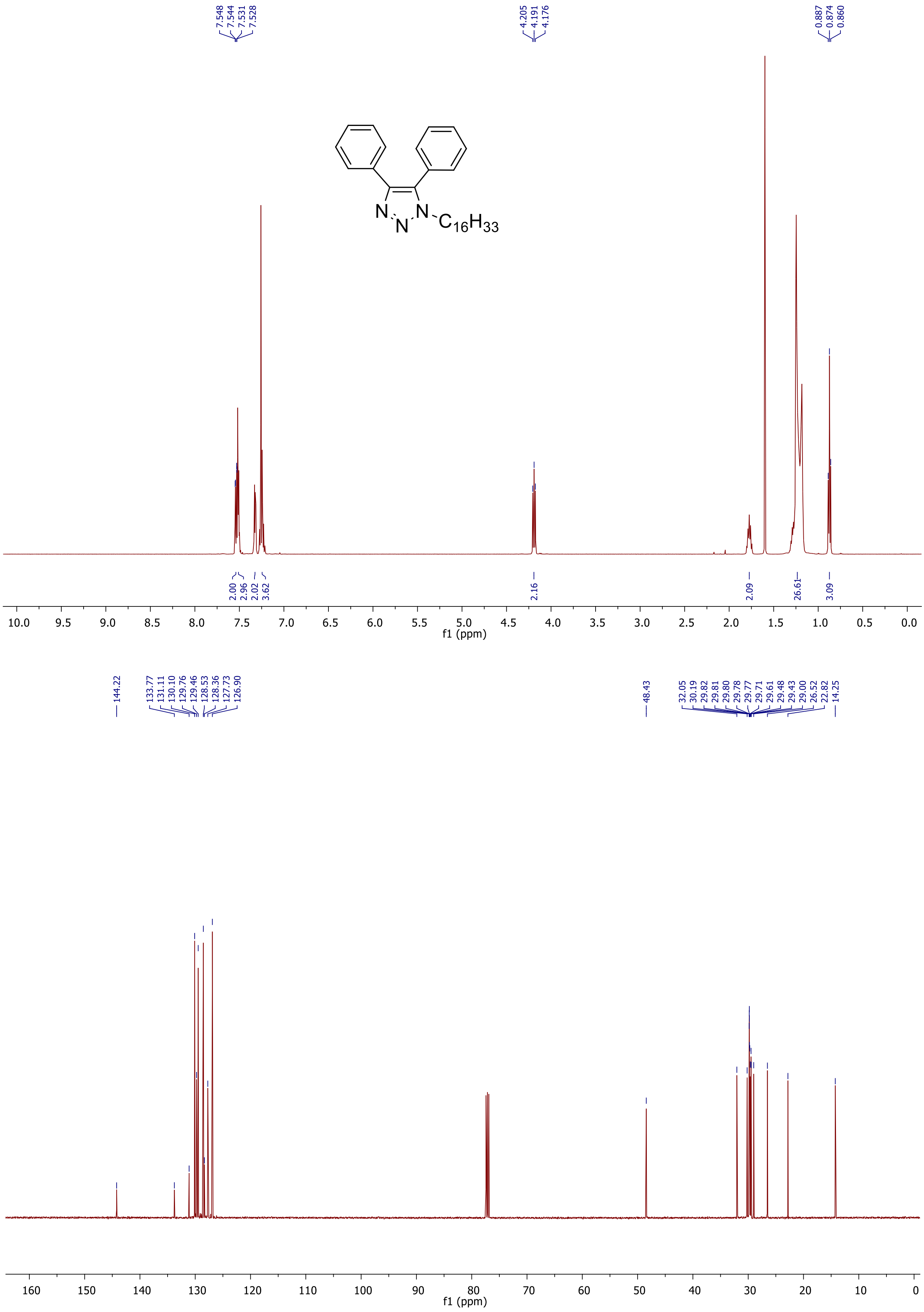

Figure S13. ^{1}H NMR and ^{13}C NMR spectra of compound **6a** (500 and 126 MHz, $CDCl_3$).

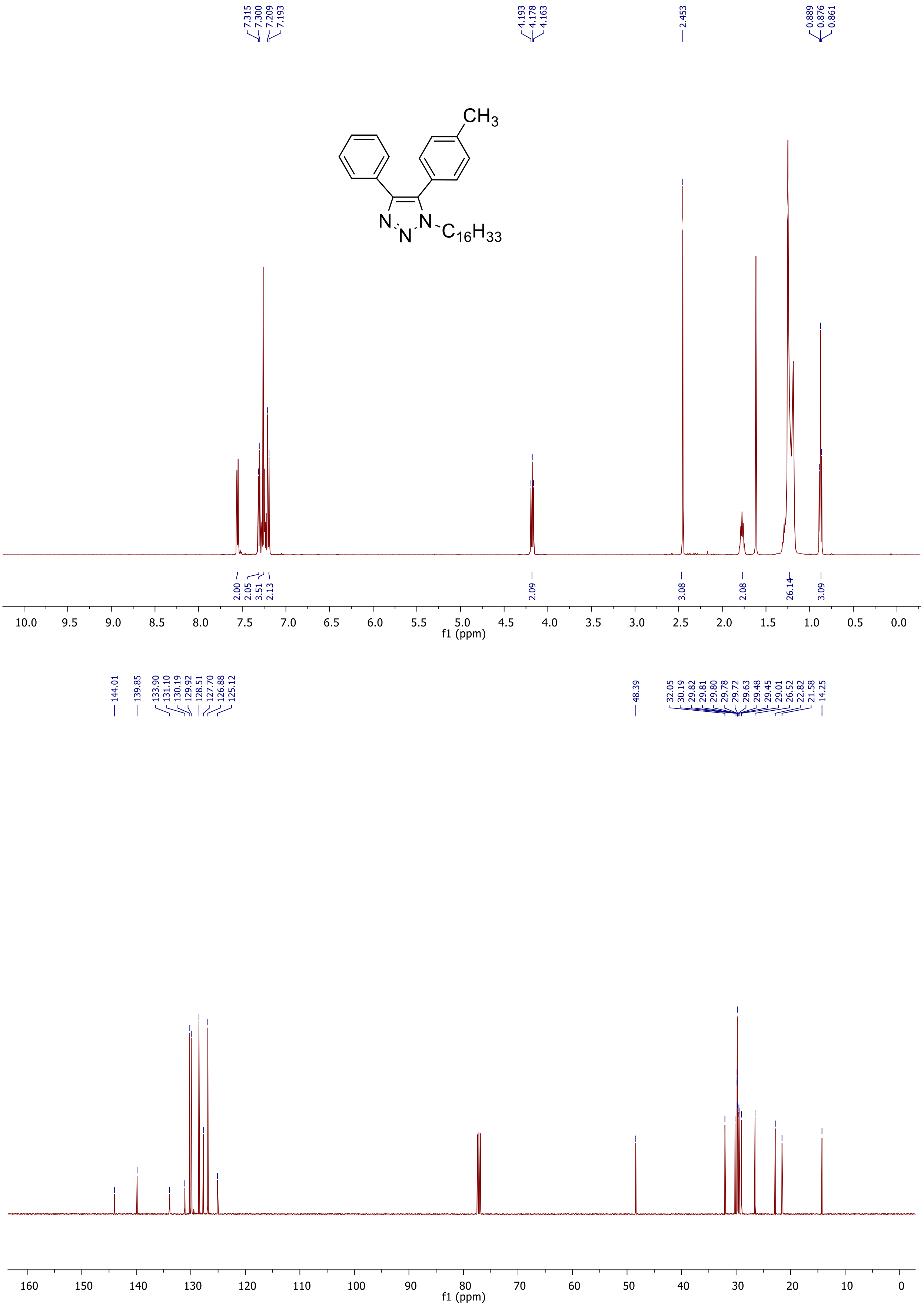

Figure S14. ^{1}H NMR and ^{13}C NMR spectra of compound **6b** (500 and 126 MHz, $CDCl_3$).

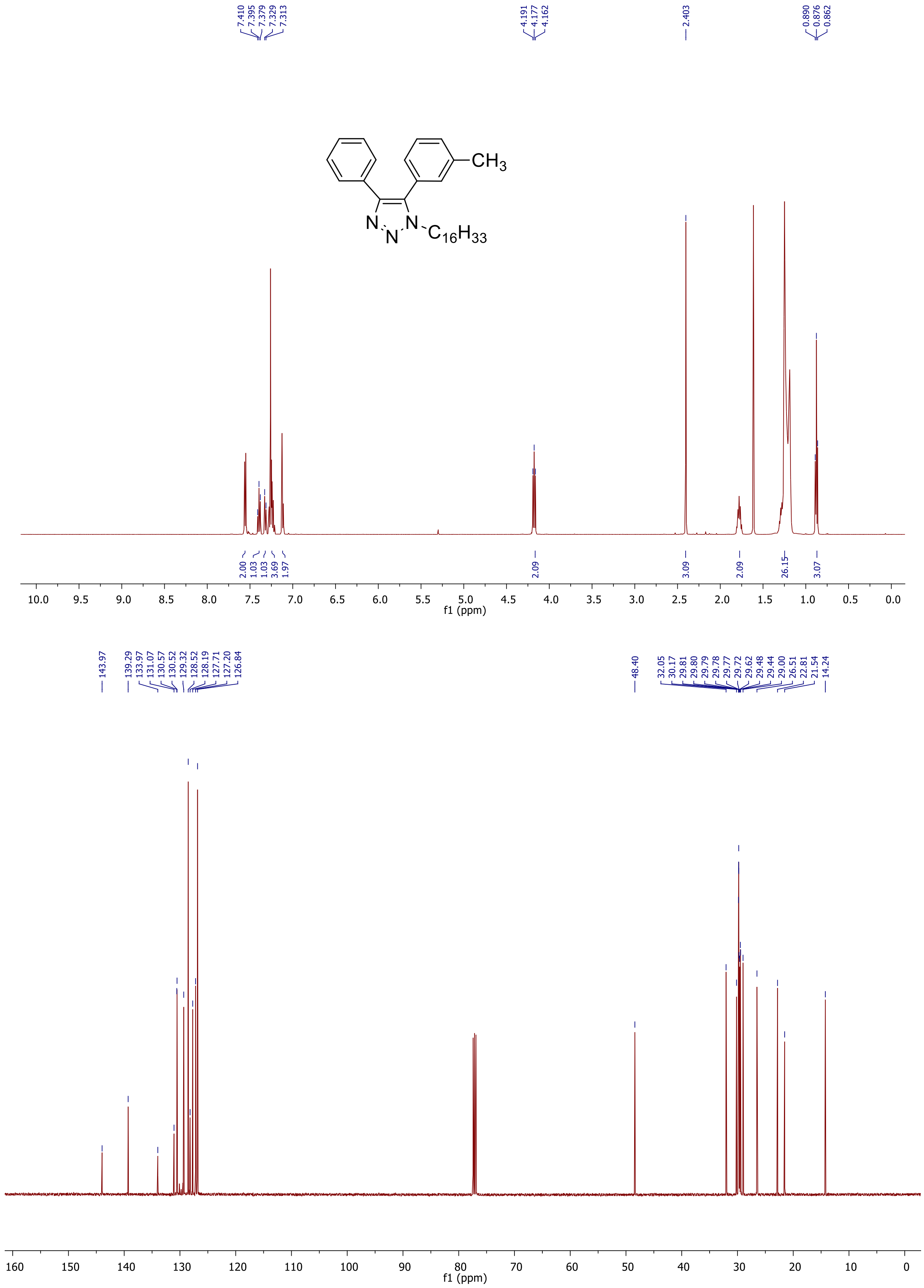

Figure S15. ^{1}H NMR and ^{13}C NMR spectra of compound **6c** (500 and 126 MHz, $CDCl_3$).

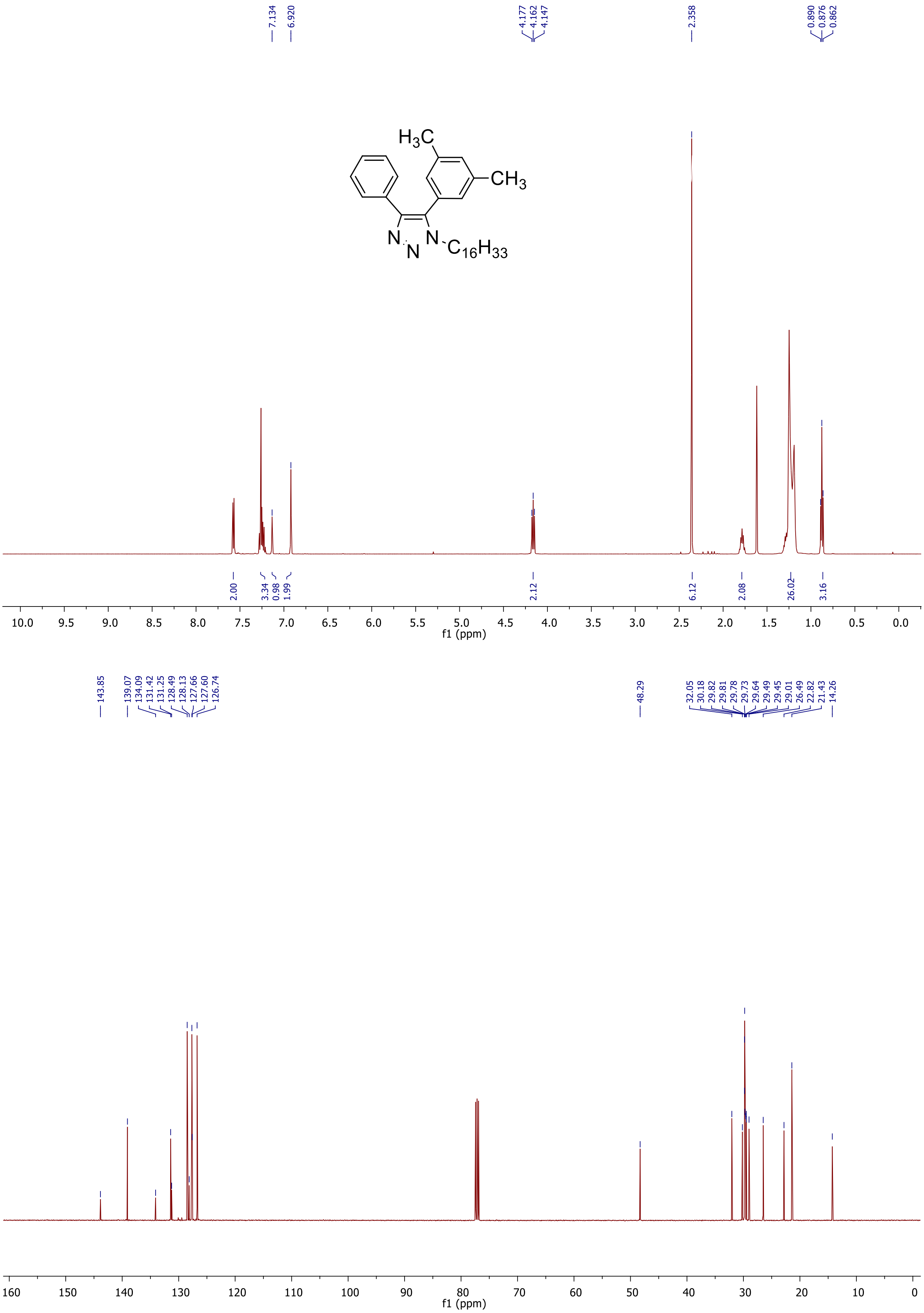

Figure S16. ^{1}H NMR and ^{13}C NMR spectra of compound **6d** (500 and 126 MHz, $CDCl_3$).

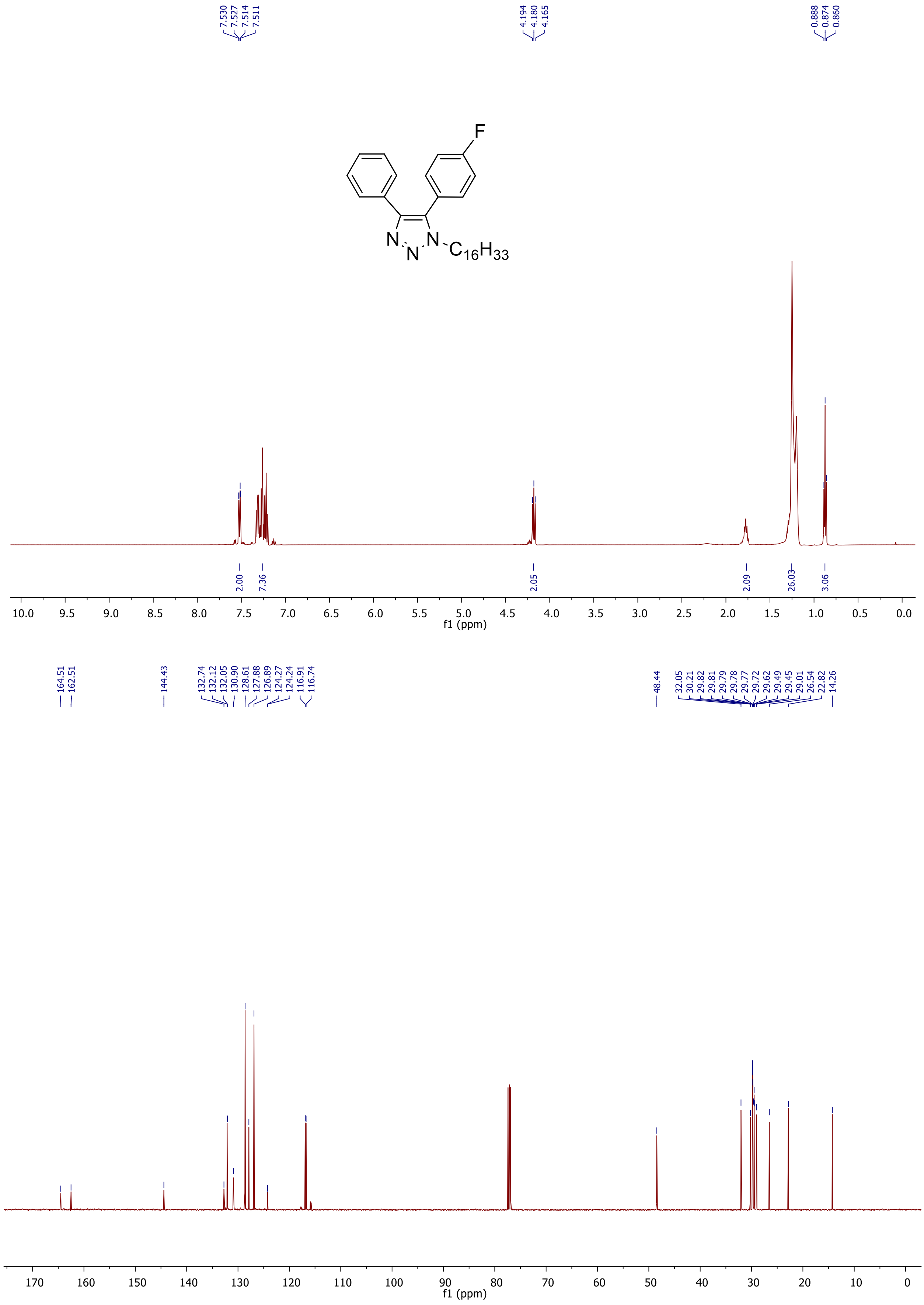

Figure S17. ^{1}H NMR and ^{13}C NMR spectra of compound **6e** (500 and 126 MHz, $CDCl_3$).

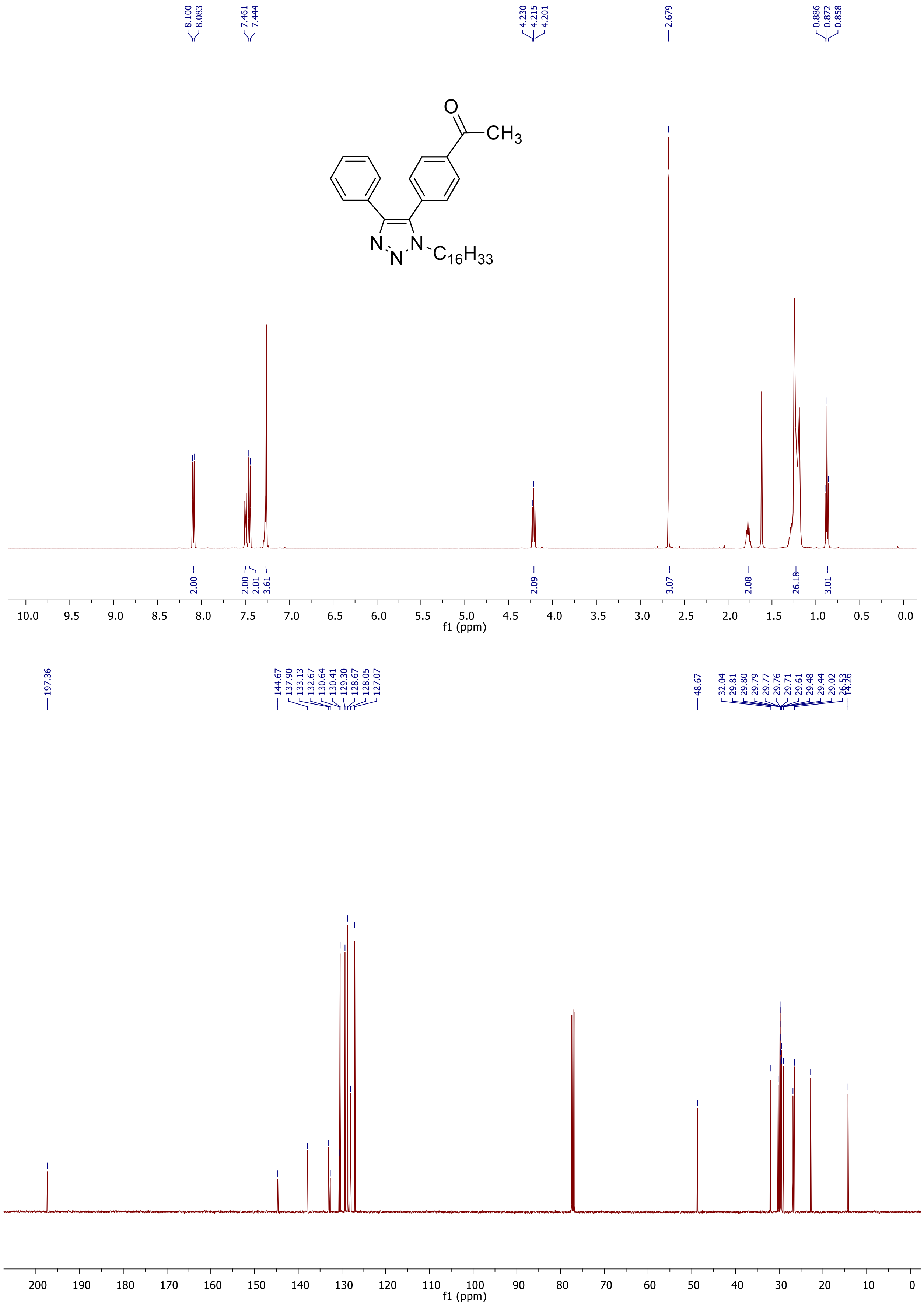

Figure S18. ^{1}H NMR and ^{13}C NMR spectra of compound **6f** (500 and 126 MHz, $CDCl_3$).

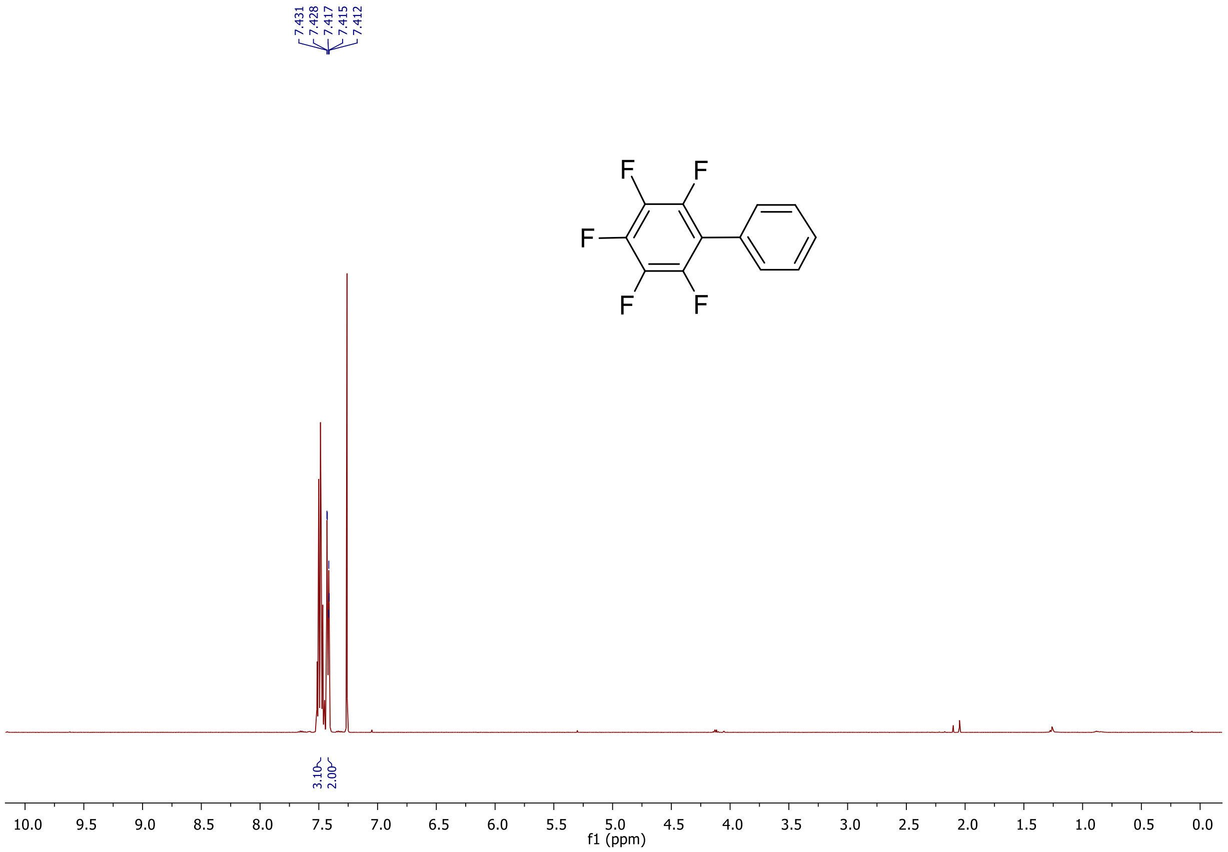

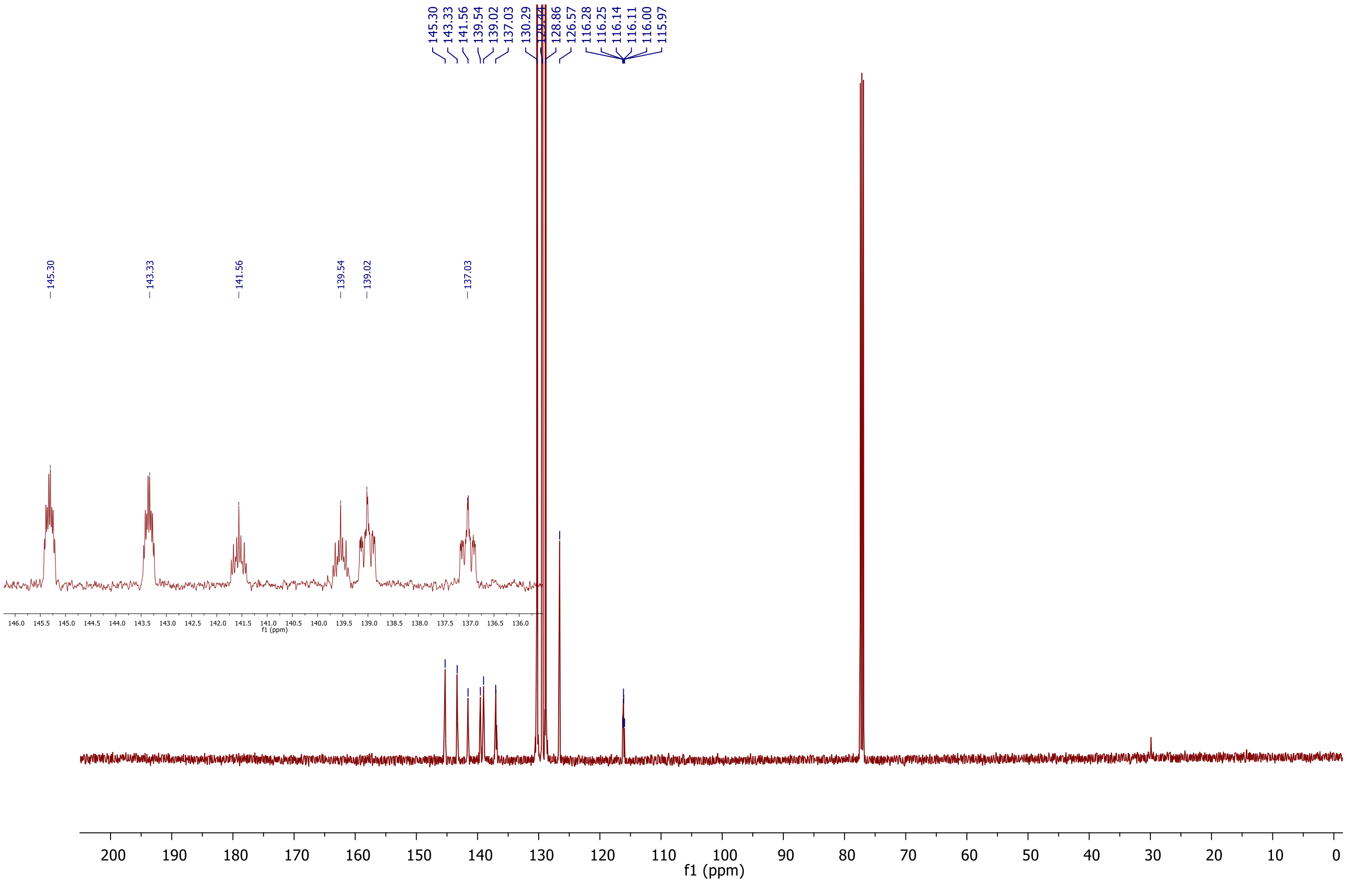

Figure S19. ^{1}H NMR and ^{13}C NMR spectra of compound **8a** (500 and 126 MHz, $CDCl_3$).

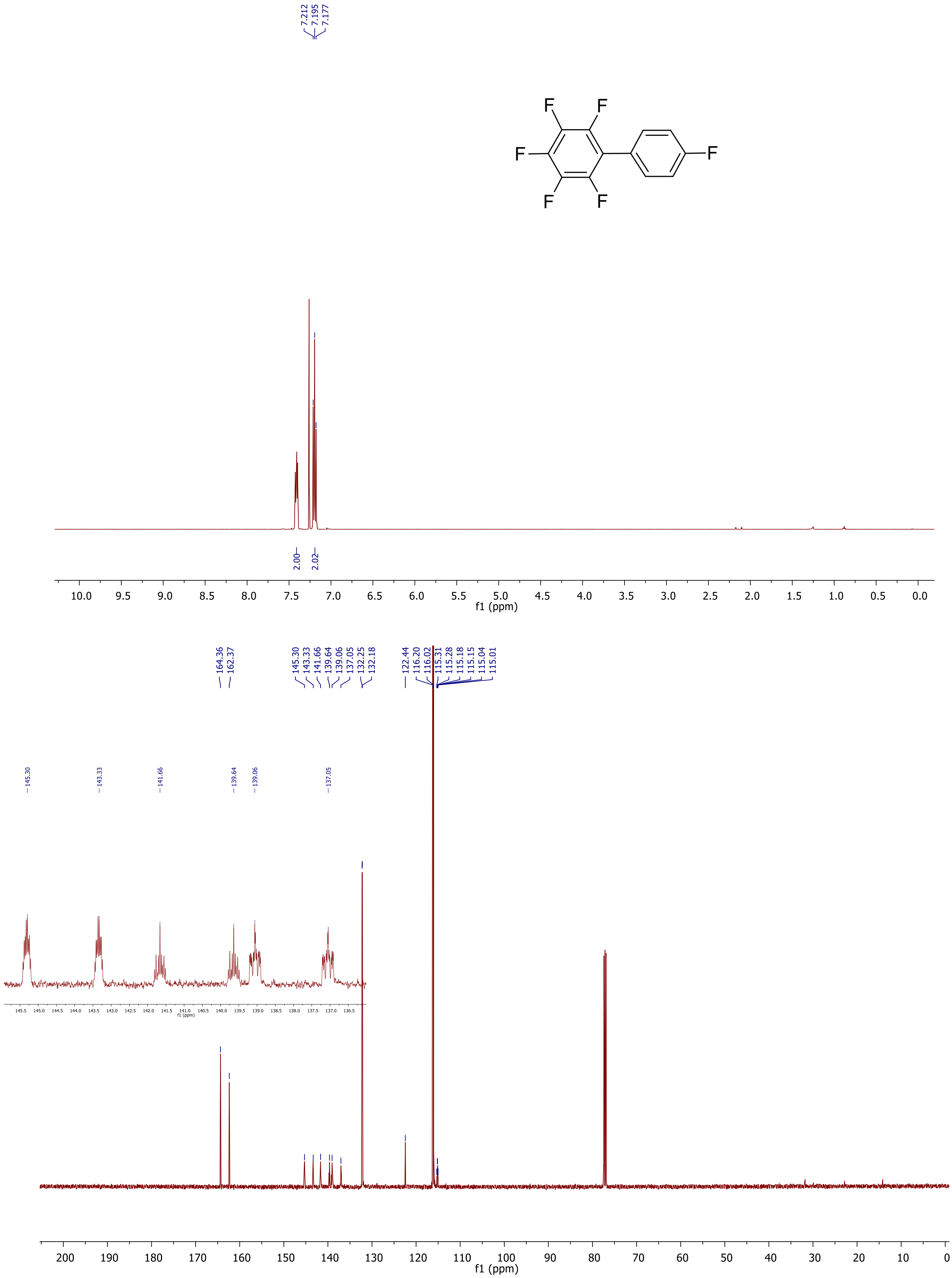

Figure S20. ^{1}H NMR and ^{13}C NMR spectra of compound **8b** (500 and 126 MHz, $CDCl_3$).

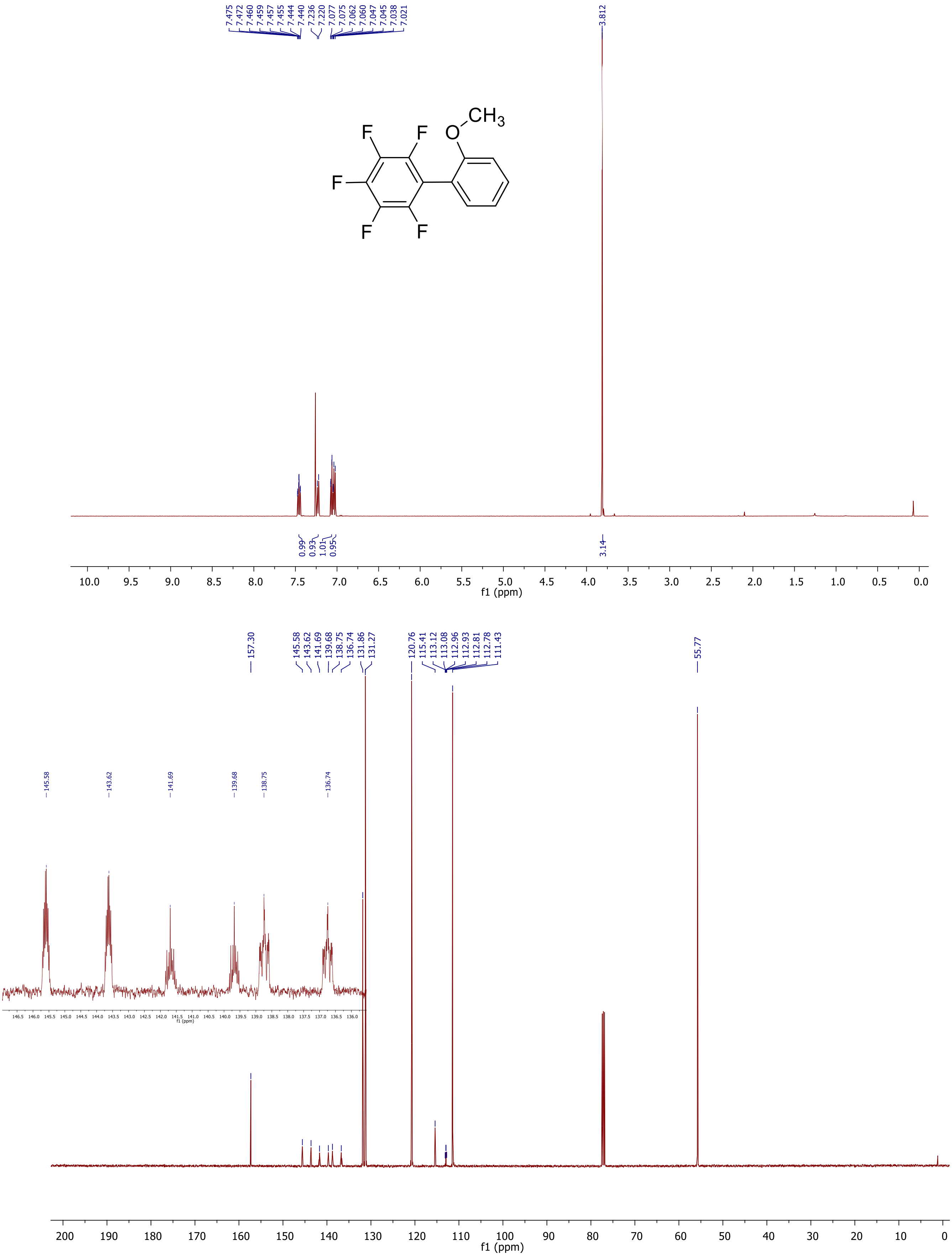

Figure S21. ^{1}H NMR and ^{13}C NMR spectra of compound **8c** (500 and 126 MHz, $CDCl_3$).

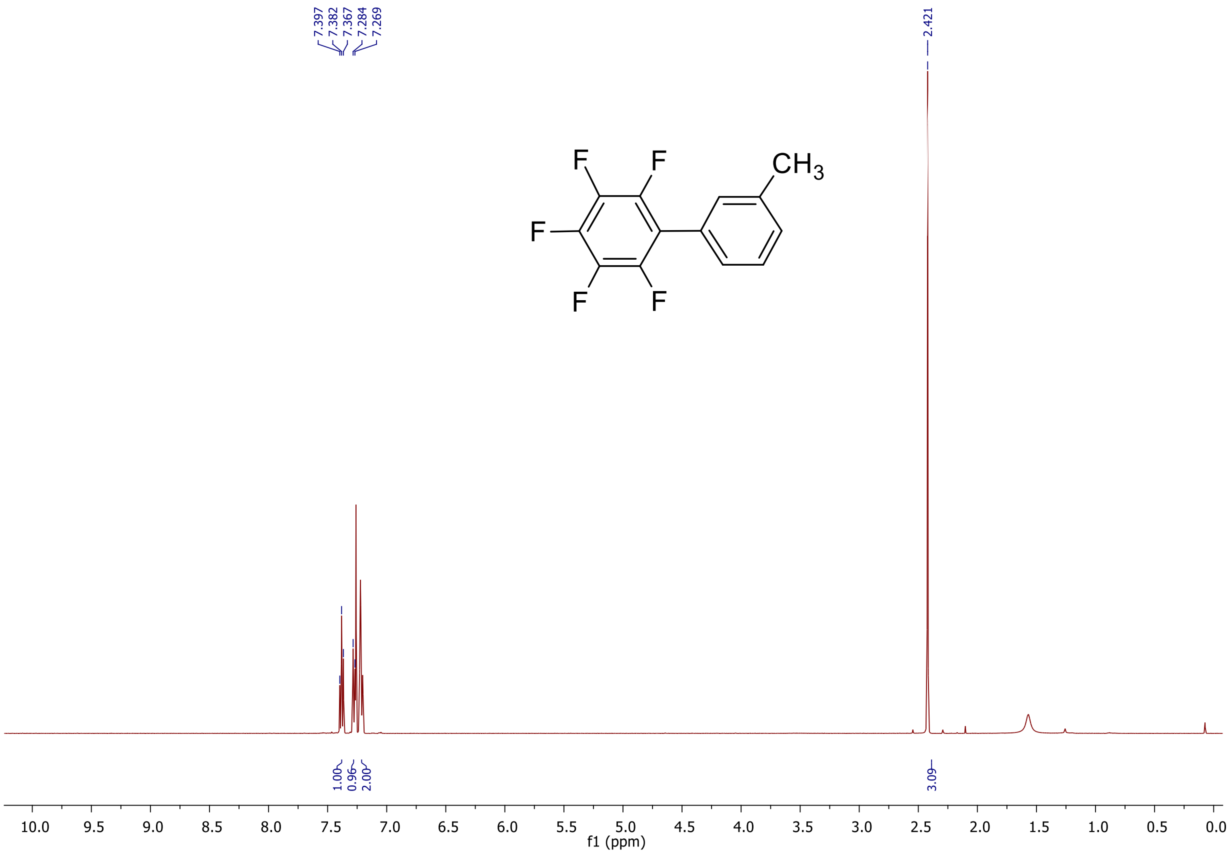

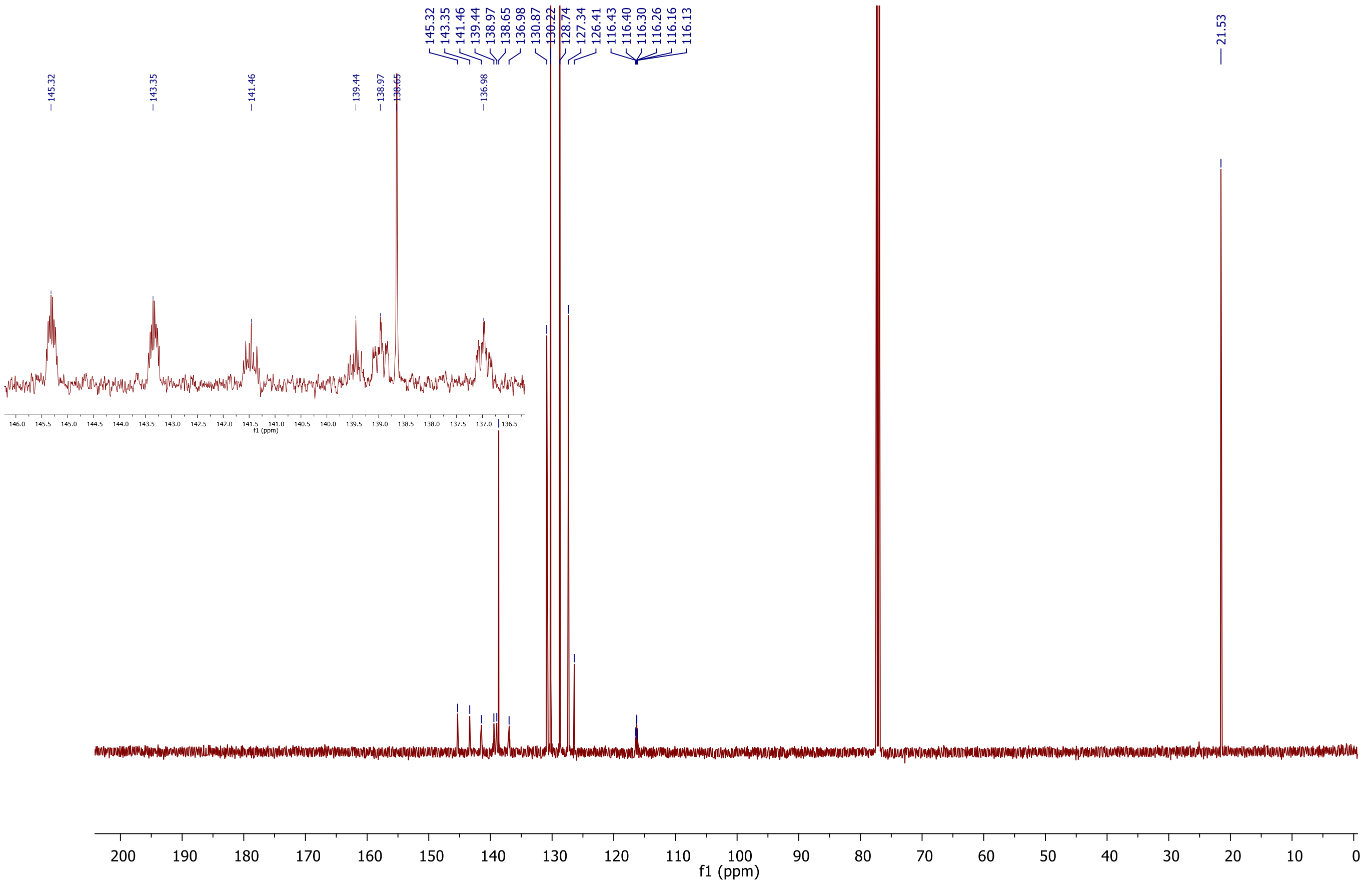

Figure S22. ^{1}H NMR and ^{13}C NMR spectra of compound **8d** (500 and 126 MHz, $CDCl_3$).

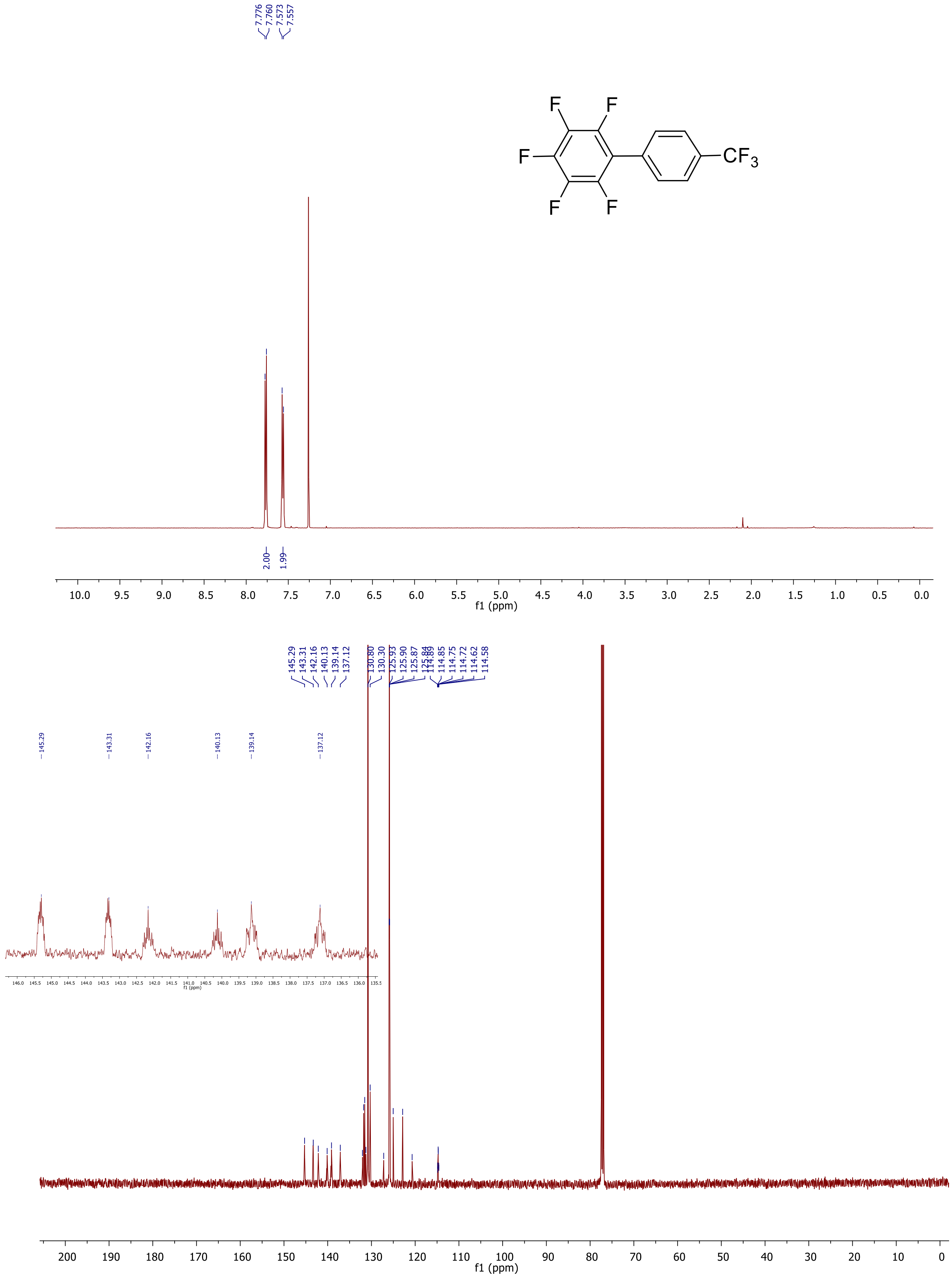

Figure S23. ^{1}H NMR and ^{13}C NMR spectra of compound **8e** (500 and 126 MHz, $CDCl_3$).

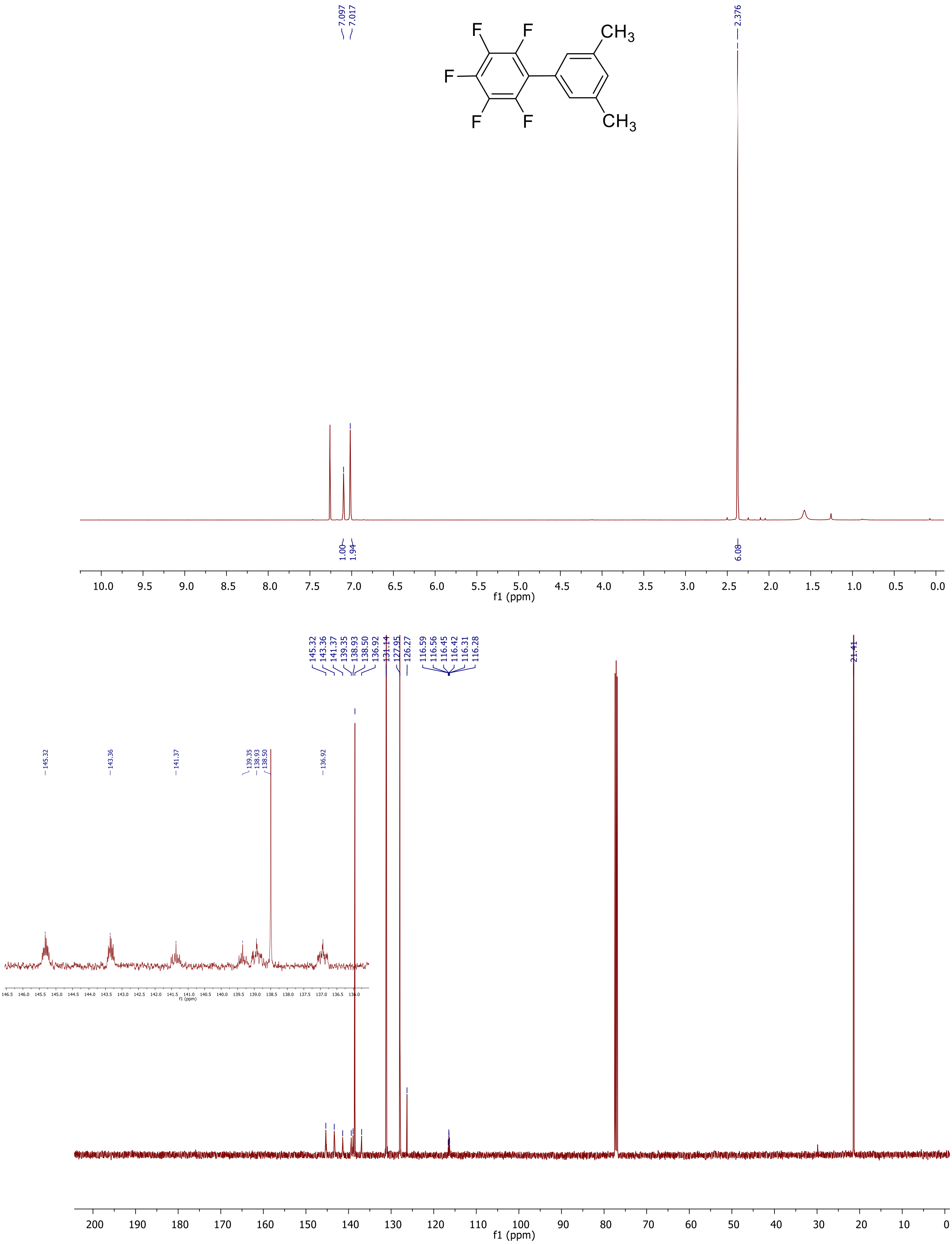

Figure S24. ^{1}H NMR and ^{13}C NMR spectra of compound **8f** (500 and 126 MHz, $CDCl_3$).

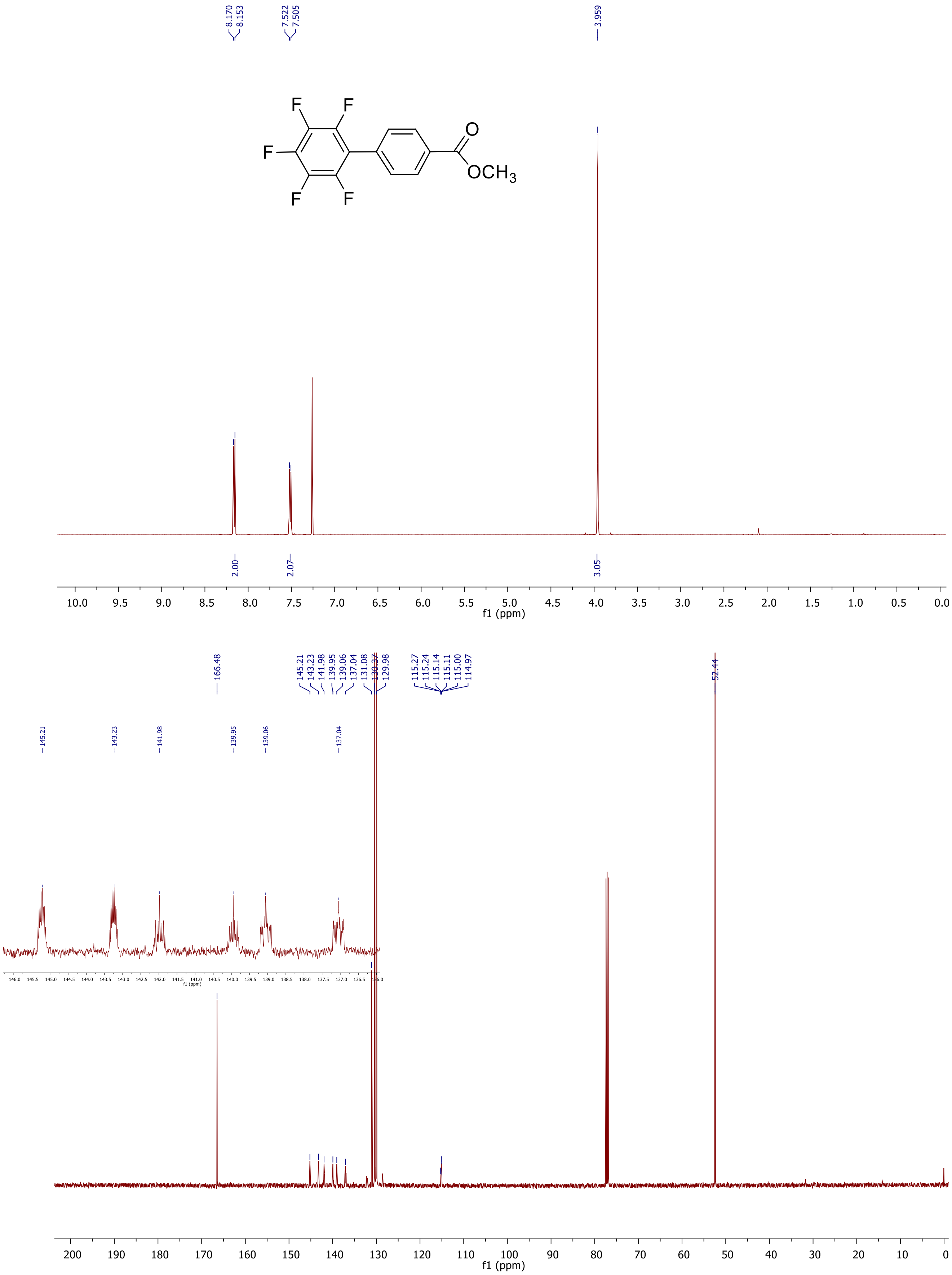

Figure S25. ^{1}H NMR and ^{13}C NMR spectra of compound **8g** (500 and 126 MHz, $CDCl_3$).

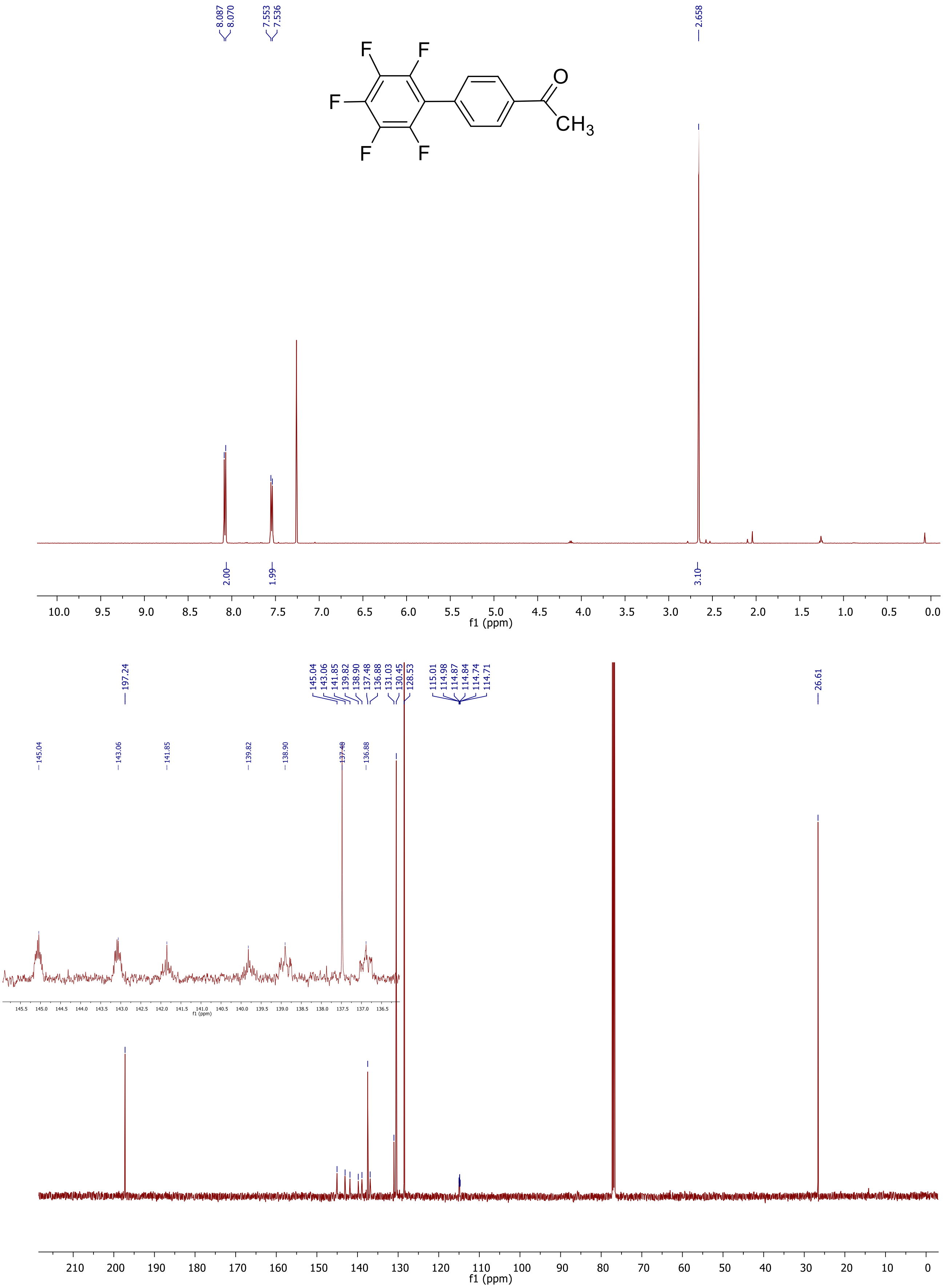

Figure S26. ^{1}H NMR and ^{13}C NMR spectra of compound **8h** (500 and 126 MHz, $CDCl_3$).

HRMS Spectra

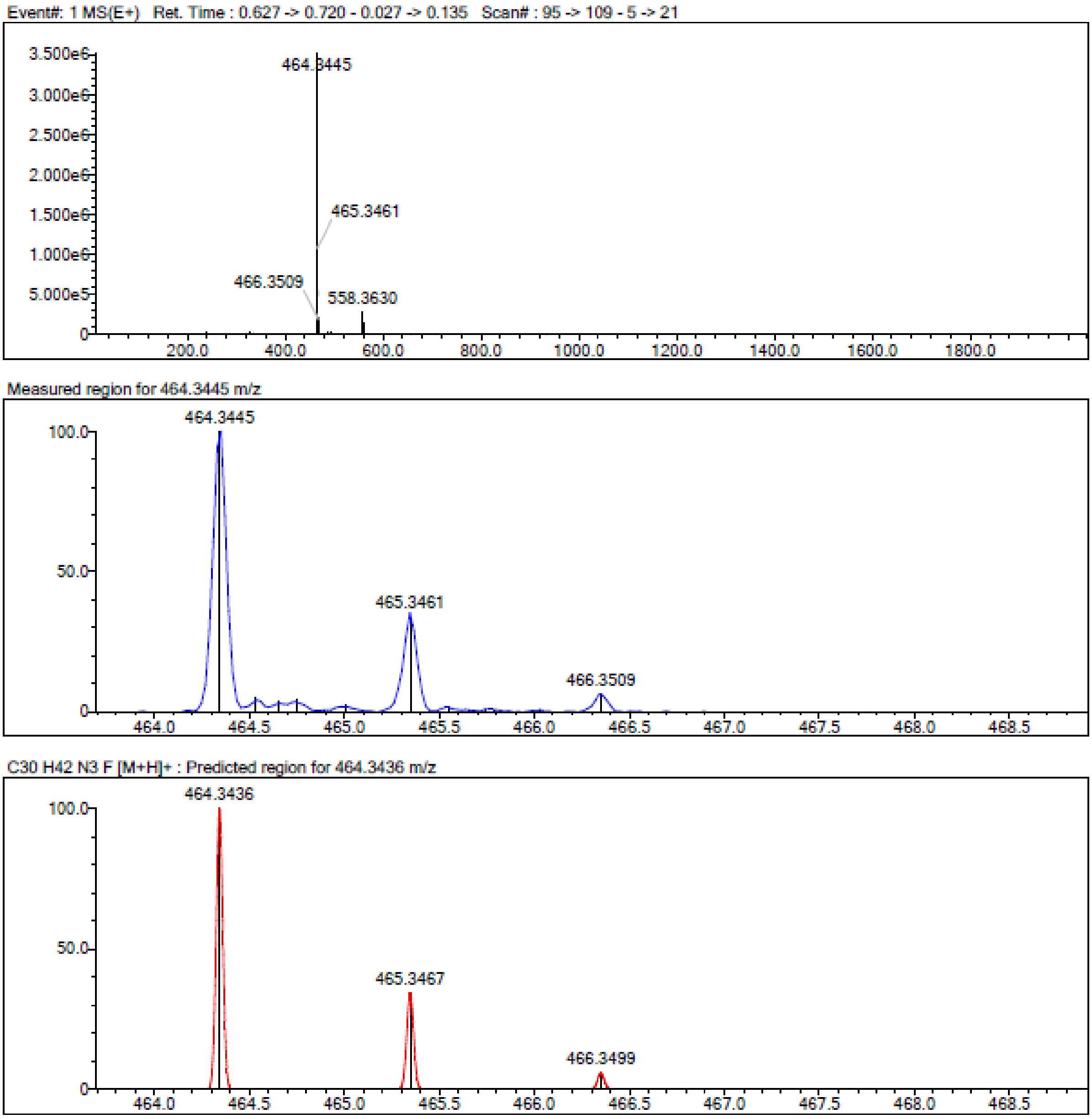

Rank	Score	Formula (M)	Ion	Meas. (M)	Pred. (M)	Meas. m/z	Pred. m/z	Df. (mDa)	Df. (ppm)	Iso	DBE
1	86.49	C30 H42 N3 F	[M+H]+	463.3372	463.3363	464.3445	464.3436	0.9	1.94	88.57	11.0

Figure S27. HRMS spectrum of compound **6e**.

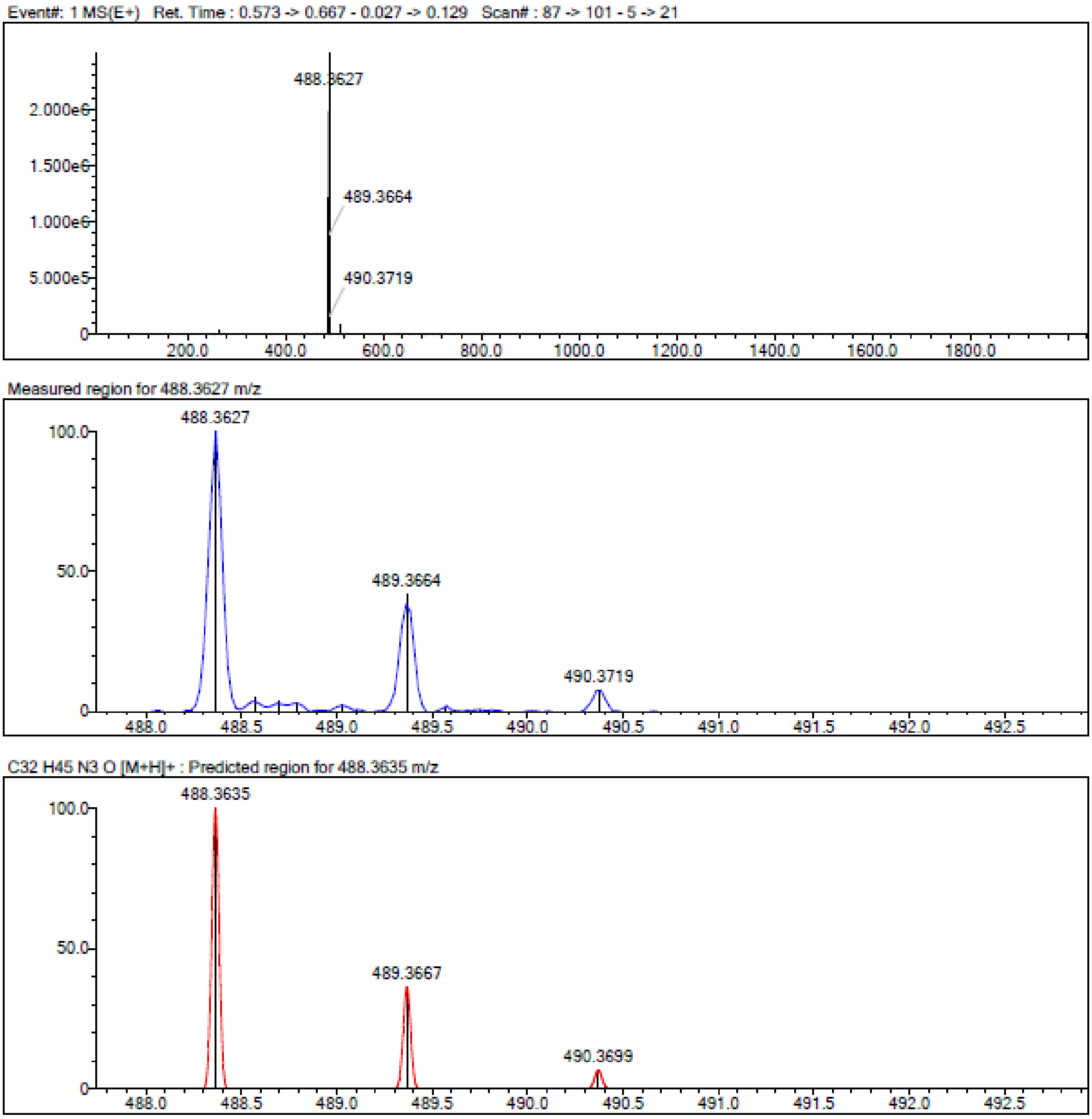

Rank	Score	Formula (M)	Ion	Meas. (M)	Pred. (M)	Meas. m/z	Pred. m/z	Df. (mDa)	Df. (ppm)	Iso	DBE
1	97.66	C32 H45 N3 O	[M+H]+	487.3554	487.3563	488.3627	488.3635	-0.8	-1.64	99.25	12.0

Figure S28. HRMS spectrum of compound **6f**.
